# Understanding the Impact of Hypoxia on Pulmonary Artery Endothelial Cells in Chronic Thromboembolic Pulmonary Hypertension Patients

**DOI:** 10.3390/ijms27073207

**Published:** 2026-04-01

**Authors:** Ylenia Roger, Anna Sardiné-Rama, Adelaida Bosacoma, Irene Gómez, Rita Fernández-Hernández, Francisco Rafael Jimenez-Trinidad, Cristina Rodríguez, Cristina Bonjoch, Isaac Almendros, Esther Marhuenda, Andrés Amalio Urrutia, Míriam Peracaula, Manuel Castellà, Isabel Blanco, Ana Ramírez, Víctor Ivo Peinado, Joan Albert Barberà, Olga Tura-Ceide

**Affiliations:** 1 Department of Pulmonary Medicine, Hospital Clínic-Institut d’Investigacions Biomèdiques August Pi i Sunyer (IDIBAPS), University of Barcelona, 08036 Barcelona, Spain; yleniaroger@gmail.com (Y.R.); sardine@recerca.clinic.cat (A.S.-R.); bosacoma@recerca.clinic.cat (A.B.); rifernandez@recerca.clinic.cat (R.F.-H.); isaac.almendros@ub.edu (I.A.); iblanco2@clinic.cat (I.B.); aramirez@clinic.cat (A.R.); vpeinado@recerca.clinic.cat (V.I.P.); 2 Biomedical Research Networking Centre on Respiratory Diseases (CIBERES), 28029 Madrid, Spain; 3 Cardiology Department, Institute Clinic Cardiovascular (ICCV), Hospital Clinic-Institute d’Investigacions Biomèdiques August Pi i Sunyer (IDIBAPS), University of Barcelona, 08036 Barcelona, Spain; franciscojimeneztrinidad@gmail.com; 4 Unitat de Biofísica i Bioenginyeria, Facultat de Medicina i Ciències de la Salut, Universitat de Barcelona, 08036 Barcelona, Spain; 5 Unidad de Investigación Hospital de Santa Cristina, Departamento de Medicina, Instituto de Investigación del Hospital Universitario La Princesa (IISP), Universidad Autónoma de Madrid, 28049 Madrid, Spain; andres.urrutia@uam.es; 6 Translational Research Group on Cardiovascular Respiratory Diseases (CAREs), Dr. Josep Trueta University Hospital de Girona, Santa Caterina Hospital de Salt and the Girona Biomedical Research Institute (IDIBGI-CERCA), 17190 Girona, Spain; mperacaula@idibgi.org; 7 Department of Cardiovascular Surgery, Institut Clínic del Tòrax, Hospital Clínic, University of Barcelona, 08036 Barcelona, Spain; mcaste@clinic.cat; 8 Department of Experimental Pathology, Institut d’Investigacions Biomèdiques de Barcelona (IIBB), Consejo Superior de Investigaciones Científicas (CSIC)–Institut d’Investigacions Biomèdiques August Pi i Sunyer (IDIBAPS) CSIC-IDIBAPS, 08036 Barcelona, Spain

**Keywords:** chronic thromboembolic pulmonary hypertension, endothelial dysfunction, hypoxia, oxidative stress

## Abstract

Pulmonary endarterectomy (PEA) specimens provide a unique source of endothelial cells (ECs) to model chronic thromboembolic pulmonary hypertension (CTEPH) in vitro. This study investigates the impact of chronic hypoxia on PEA-derived ECs, focusing on mechanisms of endothelial dysfunction and vascular remodeling. ECs from PEA specimens (EC-CTEPH) and controls were exposed to normoxia, hypoxia, and reoxygenation. Cell morphology, proliferation, migration, and expression of angiogenic and hypoxia-responsive genes were assessed. Pharmacological HIF stabilization with dimethyloxalylglycine (DMOG) was compared with hypoxia. Oxidative stress responses were evaluated using hydrogen peroxide. EC-CTEPH showed impaired adaptation to hypoxia, with reduced induction of glycolytic and angiogenic genes, altered morphology, delayed wound closure, and persistent oxidative stress after reoxygenation, consistent with defective hypoxia sensing. DMOG partially restored metabolic gene expression, indicating improved adaptation through HIF stabilization. Despite elevated basal ROS levels, oxidative challenge did not trigger adaptive glycolytic or angiogenic responses and induced distinct transcriptional profiles compared with controls. CTEPH endothelial cells display an altered response to hypoxia and oxidative stress, consistent with impaired hypoxia sensing and stress adaptation. This model highlights maladaptive endothelial features and provides a framework for future studies exploring HIF-targeted approaches in CTEPH.

## 1. Introduction

Chronic thromboembolic pulmonary hypertension (CTEPH) is a progressive pulmonary vascular disorder and a severe subtype of precapillary pulmonary hypertension (PH). It is characterized by persistent, organized pulmonary emboli (PEs) that obstruct the vascular bed of the main pulmonary arteries [1,2,3]. This obstruction impairs blood flow and increases pulmonary vascular pressure (PVR), triggering vascular remodeling that resembles the pathological features of pulmonary arterial hypertension (PAH). As a result, endothelial cells (ECs) undergo significant phenotypic transformation involving structural and functional changes [3,4].

CTEPH has an incidence of 2–6 cases/million adults, a prevalence of 26–38 cases/million adults, and a poor prognosis if untreated [5]. The gold-standard treatment for eligible patients is pulmonary endarterectomy (PEA) [4,6], a surgical procedure that removes thromboembolic material and improves hemodynamics [4]. As previously published by our group, ECs derived from PEA material serve as a unique in vitro CTEPH model to study patient-specific endothelial phenotypes and function [4,7].

Endothelial dysfunction (ED) is a key feature of CTEPH pathophysiology [7,8] together with inflammation-driven disruption of vascular homeostasis, thrombolysis, and remodeling [4,8]. This is accompanied by an imbalance between vasodilators and vasoconstrictors, leading to disruption of the nitric oxide (NO) pathway—a central regulator of vascular tone, endothelial homeostasis, and immune cell function, whose dysregulation promotes vasoconstriction, inflammation [9], and increased production of vasoconstrictors such as endotelin-1 (ET-1). Sustained vasoconstriction ultimately contributes to PH progression and heart failure if untreated [10,11].

Oxygen (O_2_) availability is an important environmental factor contributing to ED [12]. Reduced O_2_ supply has been implicated in the progression of diseases such as CTEPH [12,13]. Hypoxia-inducible factor 1α (*HIF-1α*) and 2α (*HIF-2α*) are key regulators of cellular responses to hypoxia [14,15,16,17]. They control the expression of genes involved in metabolism, angiogenesis, and vascular remodeling [18]. While *HIF-1α* is broadly expressed, *HIF-2α* is expressed mostly in ECs and plays a critical role in vascular remodeling [17]. Alterations in *HIF-2α* have been linked to pulmonary vascular disease (PVD) and PH development [17,19].

In 2017, Bochenek et al. reported an increase in *HIF-1α* and *HIF-2α* expression in ECs from PEA specimens [20,21]. These findings suggest that hypoxia contributed to endothelial, mesenchymal, and immune cell activation, promoting thrombosis in CTEPH [20]. Cells within remodeled pulmonary arteries are therefore likely exposed to a hypoxic microenvironment, which may drive ED and disease progression.

Based on this, we hypothesized that ECs from CTEPH patients exhibit an impaired adaptive response to hypoxia compared with control ECs. To test this, we used a novel hypoxia-on-a-chip system to evaluate EC responses under different O_2_ conditions and compared patient-derived cells with controls.

By characterizing molecular and cellular adaptations to hypoxia, this study aims to provide mechanistic insight into CTEPH pathophysiology and identify potential therapeutic targets.

## 2. Results

### 2.1. Rapid Hypoxia Induction Using a Novel Hypoxic Chip System

**Figure 1:**
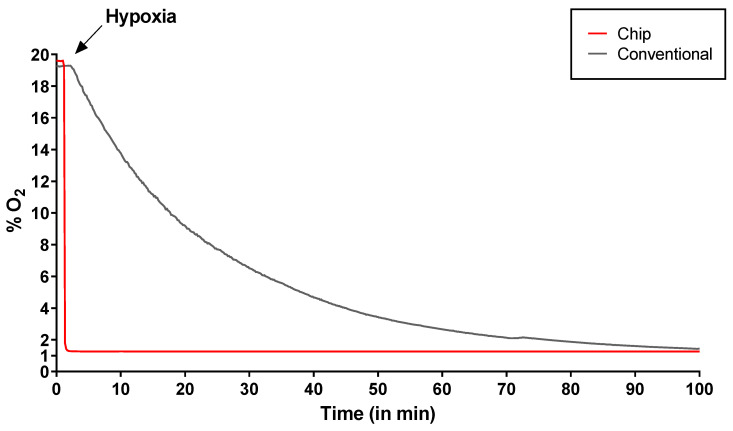
Comparison between lab-on-a-chip and conventional incubator cell culture at 1% O_2_. PO_2_ measures were taken with fiber-optic oxygen sensor (FireSting O_2_, Pyro Science, Aachen, Germany) placed above the surface of the permeable membrane, at the cell culture level. When hypoxia is induced, chip system immediately reaches 1% O_2,_ while conventional hypoxic incubator requires more than 60 min to reach 1% O_2_. *Y* axis indicates percentage of O_2,_ and *X* axis indicates time in minutes.

Oxygen pressure (PO_2_) measurements were taken with a fiber-optic oxygen sensor placed above the permeable membrane of chips and compared to PO_2_ measurements in standard cell culture dishes (Figure 1). In the new system based on chips, hypoxia was reached immediately in the liquid phase, ensuring that the cells were constantly exposed to the desired percentage of hypoxia for as long as the experiments lasted. Moreover, while chips reached 1% O_2_ as soon as the device was connected, our conventional hypoxia incubator took more than 60 min to reach 1% O_2_. The chip measurements remained stable over time (Figure 1).

### 2.2. Pulmonary Artery Endothelial Cells Present a Peak Response to 1% O_2_ at the mRNA Level In Vitro

**Figure 2:**
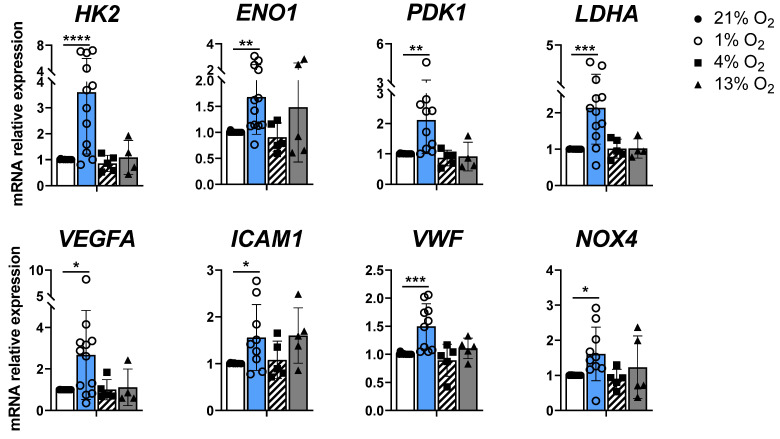
Expression of different subsets of genes in EC-Control under different O_2_ conditions. Relative mRNA expression of metabolic genes *HK2*, *ENO1*, *PDK1*, *LDHA*; angiogenic genes *VEGFA*, *ICAM1*, *VWF*; and oxidative stress gene *NOX4* in 6 independent experiments with *n* = 3 EC-Control at different passages. Statistical significance was indicated as *p* < 0.05 *, *p* < 0.01 **, *p* < 0.001 ***, *p* < 0.0001 **** (One-way ANOVA and Dunnett’s post hoc test). Each data point represents mean ± SD of relative fold change with respect to EC-Control normoxia basal levels (21% O_2_) normalized to reference gene *ACTB*.

Healthy human pulmonary artery endothelial cells (EC-Control) were cultivated under four different O_2_ culture conditions, namely, 1%, 4%, 13% and 21% O_2_, the last of which is also known as in vitro normoxia. All metabolic genes studied (*HK2*, *ENO1*, *PDK1* and *LDHA*) presented statistically significant increases in expression levels when EC-Control cells were exposed to hypoxic conditions (1% O_2_) compared with normoxic conditions (21% O_2_) (*HK2*: 3.59 ± 2.37 vs. 1.01 ± 0.01, *p* < 0.0001; *ENO1*: 1.68 ± 0.72 vs. 1.01 ± 0.01, *p* < 0.01; *PDK1*: 2.11 ± 1.10 vs. 1.01 ± 0.01, *p* < 0.01; *LDHA*: 2.14 ± 0.99 vs. 1.00 ± 0.00, *p* < 0.001) (Figure 2).

Similarly, the expression of angiogenic and oxidative stress genes (*VEGFA*, *VWF*, *ICAM1* and *NOX4*) was significantly greater at 1% O_2_ than at 21% O_2_ (*VEGFA*: 2.67 ± 2.14 vs. 1.00 ± 0.00, *p* < 0.05; *VWF*: 1.49 ± 0.40 vs. 1.01 ± 0.01, *p* < 0.001; *ICAM1*: 1.56 ± 0.70 vs. 1.01 ± 0.01, *p* < 0.05; *NOX4*: 1.61 ± 0.76 vs. 1.00 ± 0.01, *p* < 0.05) (Figure 2). The rest of the genes studied did not reach statistical significance, although *NOS3* and *SOD2* presented a tendency (Supplementary Figure S1).

Compared with normoxic cells, EC-Control cells presented no significant increase in the mRNA levels of any of the genes tested at 4% or 13% O_2_. As a result, in all subsequent hypoxia experiments, 1% O_2_ was used to define hypoxic conditions.

### 2.3. Identification of Early and Late Hypoxia-Induced Genes in the EC-Control Group

**Figure 3:**
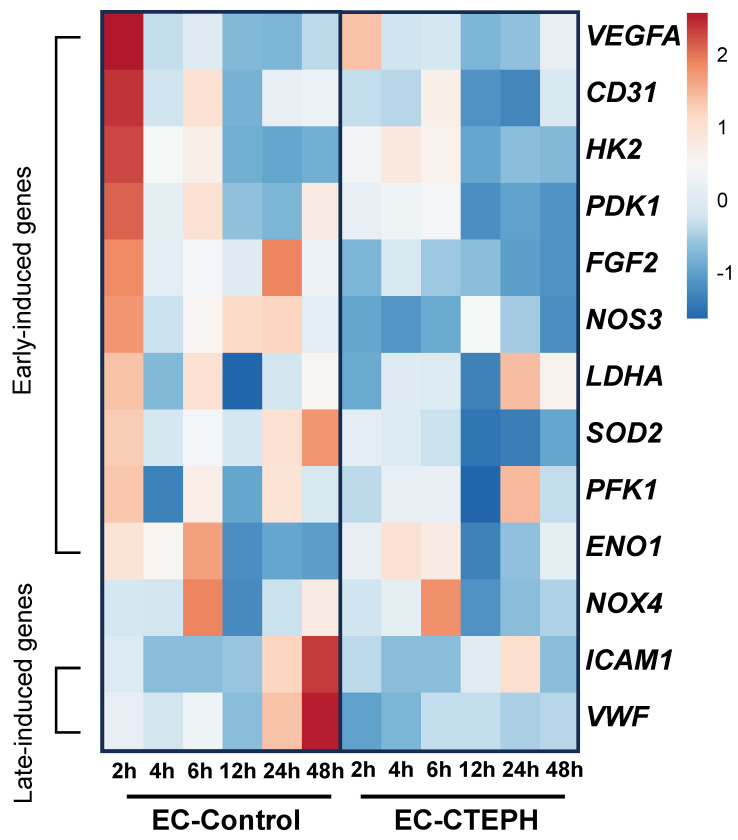
Heatmap of gene expression under hypoxia. Gene expression under 1% O_2_ (log_2_ fold-change relative to EC-Control at 21% O_2_) was measured at 2, 4, 6, 12, 24, and 48 h in three independent experiments (*n* = 3 EC-Control and *n* = 3 EC-CTEPH, passages 4–6). Data were calculated separately within each group and scaled per gene (Z-score) to visualize temporal patterns and co-regulation. Heatmap was generated using ClustVis.

EC-Control cells demonstrated a rapid induction of several genes during early hypoxia (1% O_2_, 2–6 h), with upregulation of *VEGFA*, *CD31*, *HK2*, *PDK1*, *FGF2*, *NOS3*, *LDHA*, *SOD2*, *PFK1*, and *ENO1* (Figure 3). In contrast, *ICAM1* and *VWF* were classified as late-induced genes, peaking only after 48 h under the same hypoxic conditions (1% O_2_) (Figure 3).

In EC-CTEPH cells, most genes showed minimal or no temporal changes in expression under hypoxic conditions, with a generally more uniform pattern across time points compared to EC-Control.

Figure 3 illustrates the overall gene regulation across timepoints. Statistical analyses for hypoxia vs. normoxia comparisons (per gene, per timepoint) are reported in Supplementary Table S1.

### 2.4. Effects of Hypoxia on the Morphology, Proliferation and Migration of EC-Control and EC-CTEPH Cells

**Figure 4:**
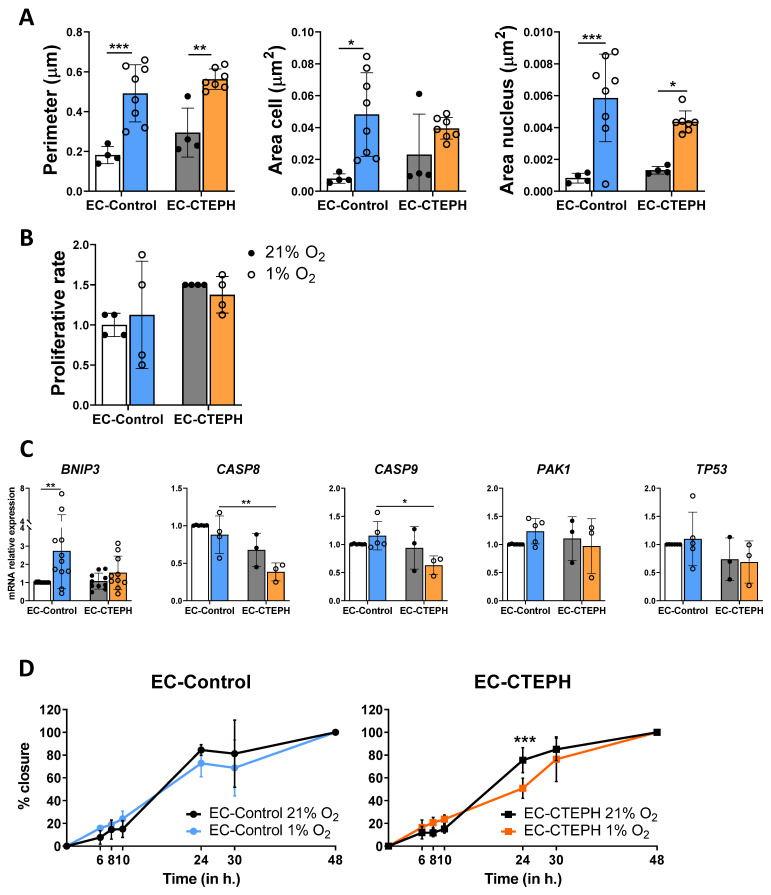
Morphology, migration and apoptotic profiles in EC-Control and EC-CTEPH under hypoxia. (**A**) Perimeter (in µm), cell area (in µm^2^) and nucleus area (in µm^2^) of EC-Control and EC-CTEPH under hypoxia and normoxia. Statistical significance was indicated as *p* < 0.05 *, *p* < 0.01 **, *p* < 0.001 *** (2-way ANOVA and Tukey’s post hoc test) (**B**) Proliferation rate of 4 independent experiments with *n* = 3 EC-Control at different passages and *n* = 4 EC-CTEPH under hypoxia and normoxia (2-way ANOVA and Tukey’s post hoc test) (**C**) mRNA relative expression of pro-apoptotic genes *BNIP3*, *CASP8*, *CASP9*, *PAK1* and *TP53.* Statistical significance was indicated as *p* < 0.05 *, *p* < 0.01 ** (2-way ANOVA and Tukey’s post hoc test). Each data point represents mean ± SD of relative fold change with respect to normoxic basal levels normalized to reference gene *ACTB*. (**D**) Migration capacity of EC-Control and EC-CTEPH in the wound healing assay, 6 independent experiments with *n* = 3 EC-Control at different passages, *n* = 5 EC-CTEPH at different passages, *p* < 0.05 ***, 2-way ANOVA and Tukey’s post hoc test, values expressed as mean ± SD. Percentage of wound closure is measured and plotted at 0, 6, 8, 10, 24, 30 and 48 h.

There was no significant difference in cellular perimeter, cellular area, or nuclear area between EC-Control and EC-CTEPH cells under normoxic conditions (Figure 4A). Under hypoxic conditions, both the perimeter and nuclear area of EC-Control cells were significantly greater than those of normoxic cells (0.492 ± 0.144 vs. 0.183 ± 0.043 and 0.006 ± 0.003 vs. 0.001 ± 0.000, *p* < 0.001, respectively). Similar increases in cell perimeter and nuclear area under hypoxia were also observed in EC-CTEPH (0.563 ± 0.051 vs. 0.294 ± 0.123, *p* < 0.01 and 0.004 ± 0.001 vs. 0.001 ± 0.000, *p* < 0.05, respectively) (Figure 4A).

The proliferation rates of both EC-Control and EC-CTEPH remained unchanged under both normoxic (1.38 ± 0.23 vs. 1.50 ± 0.00) and hypoxic conditions (1.13 ± 0.67 vs. 1.00 ± 0.14) (Figure 4B). The expression of cell cycle regulators *PAK1* and *TP53* remained stable across both normoxic and hypoxic conditions in both cell types (Figure 4C).

Under normoxia, EC-Control and EC-CTEPH cells exhibited comparable expression levels of apoptosis-related genes. Upon exposure to hypoxia, *BNIP3* was significantly upregulated in EC-Control cells (2.73 ± 2.08 vs. 1.01 ± 0.02, *p* < 0.01), whereas no such increase was detected in EC-CTEPH cells. *CASP8* and *CASP9* expression did not change in response to hypoxia in either group; however, their basal levels were consistently higher in EC-Control compared with EC-CTEPH cells (0.88 ± 0.25 vs. 0.38 ± 0.12, *p* < 0.01, and 1.15 ± 0.25 vs. 0.63 ± 0.17, *p* < 0.05, respectively) (Figure 4C).

Cellular migration, as measured by the wound healing assay, revealed that both EC-Control and EC-CTEPH groups achieved 100% wound closure at 48 h under both normoxic and hypoxic conditions. However, compared with normoxia, EC-CTEPH displayed a significantly delayed wound closure under hypoxia at the 24 h mark, which was not observed in the EC-Control group. The average degree of wound closure in EC-CTEPH under hypoxia was 50.88% ± 8.95, compared with 75.54% ± 11.04 under normoxia (*p* < 0.001) (Figure 4D). As shown in Supplementary Table S5, at 24 h, EC-CTEPH cells exposed to hypoxia (1% O_2_) showed significantly lower wound closure compared with both their normoxic counterparts (21% O_2_) and EC-Control cells under hypoxic conditions.

### 2.5. Differential Gene Expression Analysis Reveals a Significant Hypoxic Response in EC-CTEPH Compared with EC-Control

**Figure 5:**
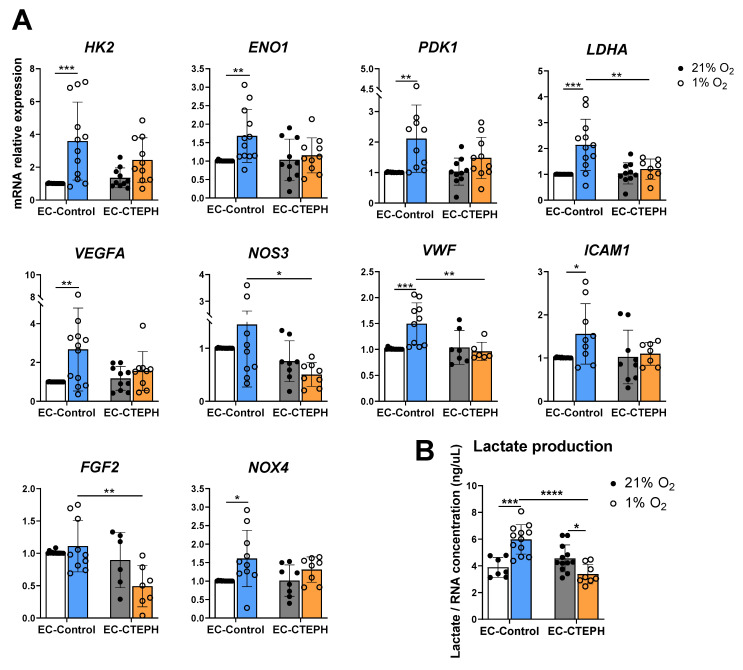
Expression of different subsets of genes in EC-Control and EC-CTEPH under hypoxia. (**A**) Relative mRNA expression of metabolic genes *HK2*, *ENO1*, *PDK1*, *LDHA*, angiogenic genes *VEGFA*, *NOS3*, *VWF*, *ICAM1*, *FGF2*; and oxidative stress gene *NOX4* in six independent experiments (*n* = 3 EC-Control and *n* = 5 EC-CTEPH, passages 4–6). Each data point represents mean ± SD of relative fold change compared to EC-Control normoxic basal levels (21% O_2_), normalized to reference gene *ACTB.* Statistical significance was indicated as *p* < 0.05 *, *p* < 0.01 **, *p* < 0.001 *** (2-way ANOVA followed by Tukey’s post hoc test). (**B**) Lactate released into culture medium, normalized by RNA concentration (ng/µL) in six independent experiments (*n* = 3 EC-Control and *n* = 5 EC-CTEPH, passages 4–6) under hypoxia and normoxia. Values expressed as mean ± SD. Statistical significance: *p* < 0.05 *, *p* < 0.001 ***, *p* < 0.0001 **** (2-way ANOVA followed by Tukey’s post hoc test).

EC-Control significantly increased the expression levels of the metabolic glycolytic genes *HK2*, *ENO1*, *PDK1* and *LDHA* when induced by hypoxia (Figure 5A). *HK2* expression increased 3.5-fold in the EC-Control group under hypoxia, 1.7-fold in *ENO1*, and 2.1-fold in both *PDK1* and *LDHA* (Figure 5A). Accordingly, lactate levels—the terminal metabolite of glycolysis—were significantly elevated in the supernatant of the EC-Control group under hypoxic conditions compared with normoxia (5.98 ± 1.10 vs. 3.89 ± 0.75, *p* < 0.01) (Figure 5B).

**Figure 6:**
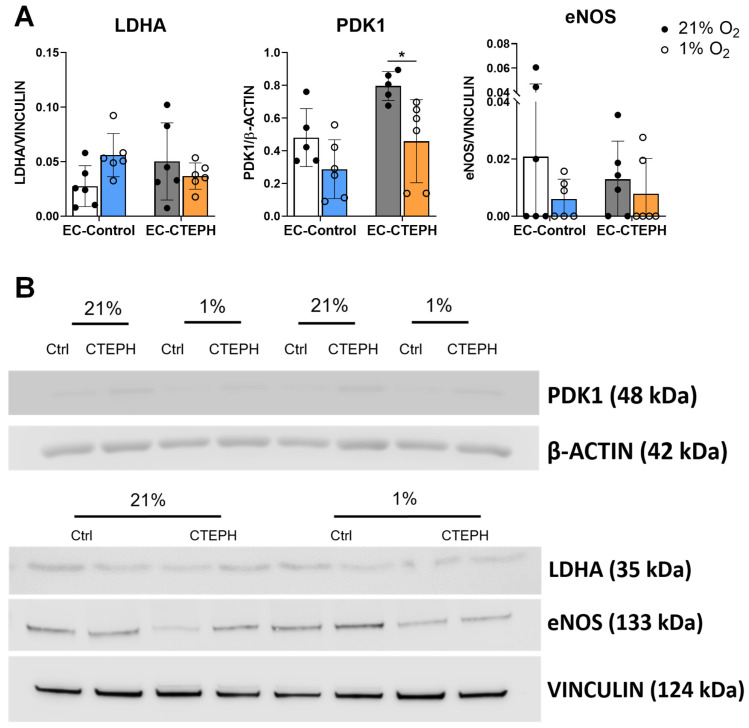
Expression of different proteins in EC-Control and EC-CTEPH under hypoxia. (**A**) Protein expression of LDHA, PDK1 and NOS3 in EC-Control and EC-CTEPH (*n* = 3 per group). Each data point represents the mean ± SD of relative fold change normalized to the reference proteins β-ACTIN and VINCULIN. Statistical significance: *p* < 0.05 * (2-way ANOVA followed by Tukey’s post hoc test). (**B**) Western blot images showing detection of target proteins PDK1, LDHA and eNOS and their respective loading controls (β-ACTIN or VINCULIN) in EC-Control and EC-CTEPH under normoxic and hypoxic conditions.

Interestingly, EC-CTEPH cells failed to significantly upregulate the expression of the previously described glycolytic genes. Consistently, lactate levels were reduced rather than increased under hypoxic conditions (3.38 ± 0.73 vs. 4.56 ± 1.01, *p* < 0.05) (Figure 5B), and hypoxia did not induce accumulation of lactate dehydrogenase A (LDHA) protein (Figure 6A,B).

Compared with those in normoxia, the mRNA levels of several angiogenic genes in EC-Control also increased under hypoxia (Figure 5A). Specifically, *VEGFA* levels increased 2.7-fold, and *VWF* and *ICAM1* levels increased 1.5-fold. *NOX4* expression levels were also increased in the EC-Control group under hypoxia compared with those under normoxia (1.6-fold) (Figure 5A). In contrast, EC-CTEPH cells did not show any significant upregulation of these genes under hypoxia, unlike EC-Control cells. In addition, the expressions of several angiogenic genes were significantly reduced between the EC-Control and EC-CTEPH groups under hypoxia (*NOS3*: 1.45 ± 1.18 vs. 0.51 ± 0.22, *p* < 0.05; *VWF*: 1.49 ± 0.40 vs. 0.96 ± 0.18, *p* < 0.01; and *FGF2*: 1.11 ± 0.39 vs. 0.49 ± 0.32, *p* < 0.01) (Figure 5A). The expression levels of *PFK1*, *CD31* and *SOD2* remained unchanged in both EC-Control and EC-CTEPH cells under hypoxic conditions (Supplementary Figure S2).

At the protein level, LDHA and eNOS showed no significant differences between conditions or groups, whereas PDK1 protein levels were significantly reduced in EC-CTEPH under hypoxia compared with normoxia (Figure 6A,B).

### 2.6. Reoxygenation Reverses the Upregulation of Genes in EC-Control Cells, but Not in EC-CTEPH Cells

**Figure 7:**
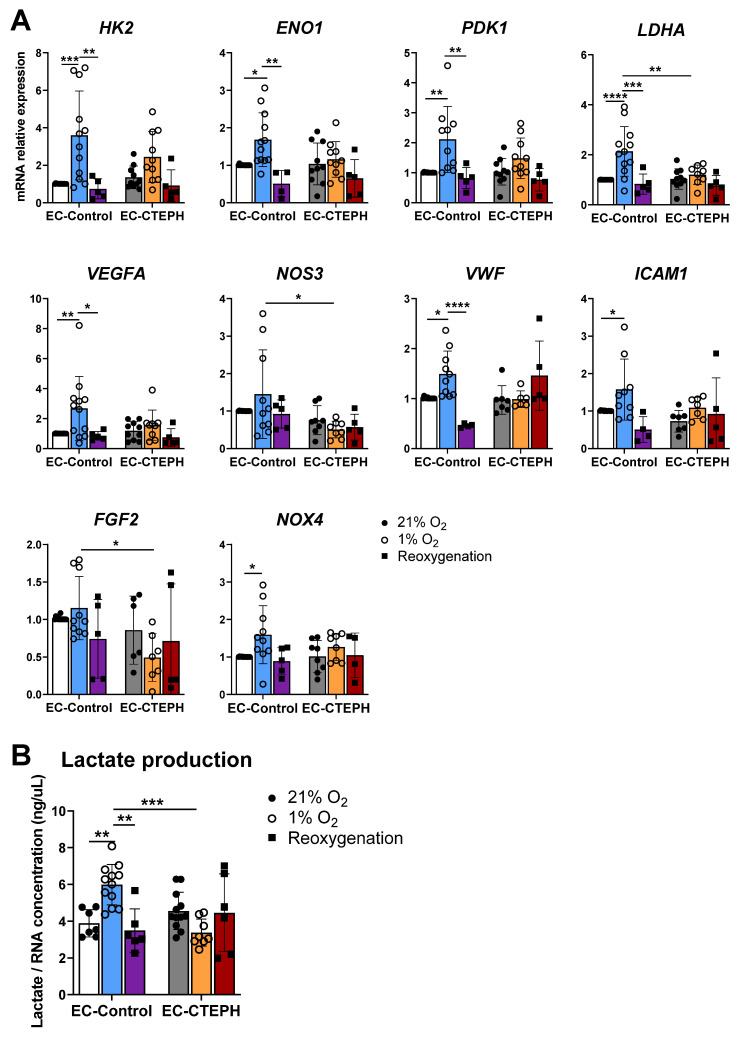
Expression of different subsets of genes in EC-Control and EC-CTEPH under hypoxia and reoxygenation. (**A**) Relative mRNA expression of metabolic genes *HK2*, *ENO1*, *PDK1*, *LDHA*; angiogenic genes *VEGFA*, *NOS3*, *VWF*, *ICAM1*, *FGF2*; and oxidative stress gene *NOX4* in 6 independent experiments with *n* = 3 EC-Control at different passages and *n* = 5 EC-CTEPH at different passages. Statistical significance was indicated as *p* < 0.05 *, *p* < 0.01 **, *p* < 0.001 ***, *p* < 0.0001 **** (2-way ANOVA and Tukey’s post hoc test). Each data point represents mean ± SD of relative fold change with respect to EC-Control normoxia basal levels (21% O_2_) normalized to reference gene *ACTB*. (**B**) Lactate released in cellular media normalized by RNA concentration in ng/µL in 6 independent experiments with *n* = 3 EC-Control and *n* = 5 EC-CTEPH, both in hypoxia, normoxia and reoxygenation, *p* < 0.01 **, *p* < 0.001 ***, 2-way ANOVA and Tukey’s post hoc test. Values expressed as mean ± SD.

In EC-Control cells, hypoxia-induced upregulation of metabolic genes returned to normoxic levels after reoxygenation (*HK2*: 3.59 ± 2.37 vs. 0.74 ± 0.53, *p* < 0.01; *ENO1*: 1.68 ± 0.72 vs. 0.51 ± 0.35, *p* < 0.01; *PDK1*: 2.11 ± 1.10 vs. 0.82 ± 0.35, *p* < 0.01; and *LDHA*: 2.14 ± 0.99 vs. 0.83 ± 0.40, *p* < 0.001) (Figure 7A), consistent with lactate levels normalizing post-reoxygenation (0.83 ± 0.40 vs. 2.14 ± 0.99, *p* < 0.01) (Figure 7B). In contrast, reoxygenation had no effect on gene expression or lactate levels in EC-CTEPH cells (Figure 7A,B).

Similarly, the significant increase in angiogenic gene expression observed under hypoxia normalized to baseline levels following reoxygenation in EC-Control cells (Figure 7A) (*VEGFA*: 2.67 ± 2.14 vs. 0.85 ± 0.29, *p* < 0.05; *VWF*: 1.49 ± 0.46 vs. 0.460 ± 0.04, *p* < 0.0001). No effect was observed with EC-CTEPH.

### 2.7. HIF Pathway Activation Occurs Both in EC-Control and EC-CTEPH

**Figure 8:**
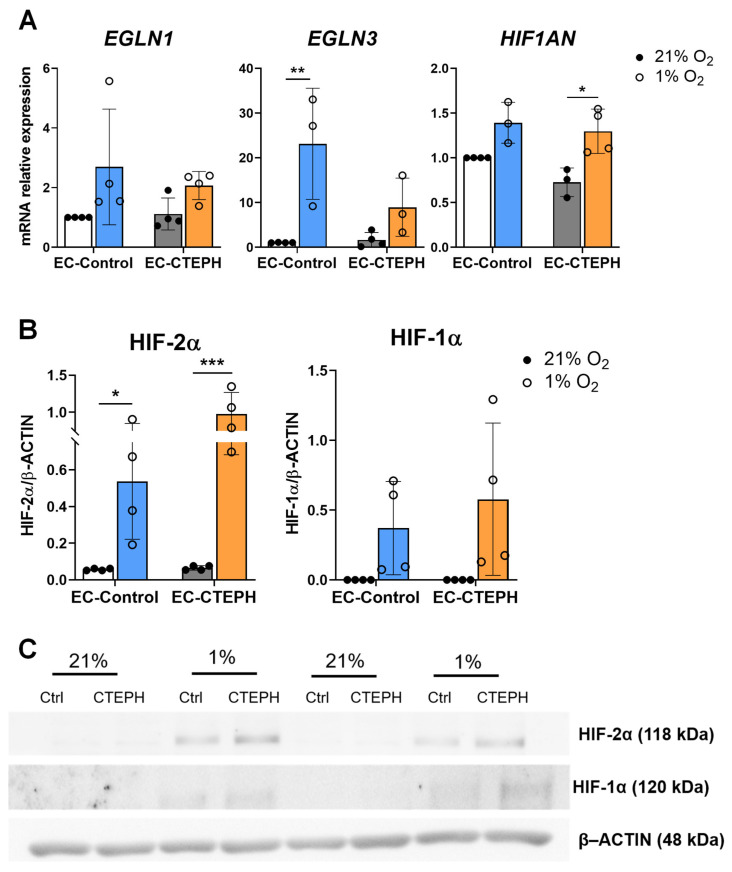
Expression of genes and proteins in HIF signaling pathway in EC-Control and EC-CTEPH under hypoxia. (**A**) mRNA relative expression of genes *EGLN1*, *EGLN3* and *HIF1AN* in 4 independent experiments with *n* = 3 EC-Control and *n* = 3 EC-CTEPH. Statistical significance was indicated as *p* < 0.05 *, *p* < 0.01 ** (2-way ANOVA and Tukey’s post hoc test). Each data point represents mean ± SD of relative fold change with respect to EC-Control normoxia basal levels (21% O_2_) normalized to reference gene *ACTB*. (**B**) Protein expression of HIF-2α and HIF-1α in four independent experiments (*n* = 3 EC-Control and *n* = 4 EC-CTEPH, passages 4–6). Each data point represents mean ± SD of relative fold change normalized to reference protein β-ACTIN. Representative blots are shown from four independent experiments. Quantification was performed by densitometry. Statistical significance was indicated as *p* < 0.05 *, *p <* 0.001 *** (2-way ANOVA and Tukey’s post hoc test). (**C**) Western blot images showing detection of target proteins HIF-2α and HIF-1α and their loading control β-ACTIN in EC-Control and EC-CTEPH under normoxic and hypoxic conditions.

Compared with normoxia, EC-Control significantly upregulated the expression of the prolyl hydroxylase 3 *EGLN3* under hypoxia. EC-CTEPH also showed a non-significant trend toward an increase in *EGLN3* expression (23.13 ± 12.43 vs. 1.05 ± 0.04). In addition, expression of the asparaginyl hydroxylase *HIF1AN* was significantly increased in EC-CTEPH under hypoxia (1.29 ± 0.25 vs. 0.73 ± 0.16, *p* < 0.05), with a similar but non-significant trend observed in EC-Control cells (Figure 8A). At the protein level, both HIF-1α and HIF-2α were upregulated under hypoxia in both EC-Control and EC-CTEPH cells, with no significant differences between groups. Notably, HIF-2α protein levels were significantly higher than HIF-1α in both cell types, suggesting predominant activation of HIF-2-dependent signaling under hypoxic conditions (Figure 8B,C).

### 2.8. Administration of Dimethyloxalylglycine (DMOG) Induced the Upregulation of Hypoxia Target Genes in Both the EC-Control and EC-CTEPH Groups

**Figure 9:**
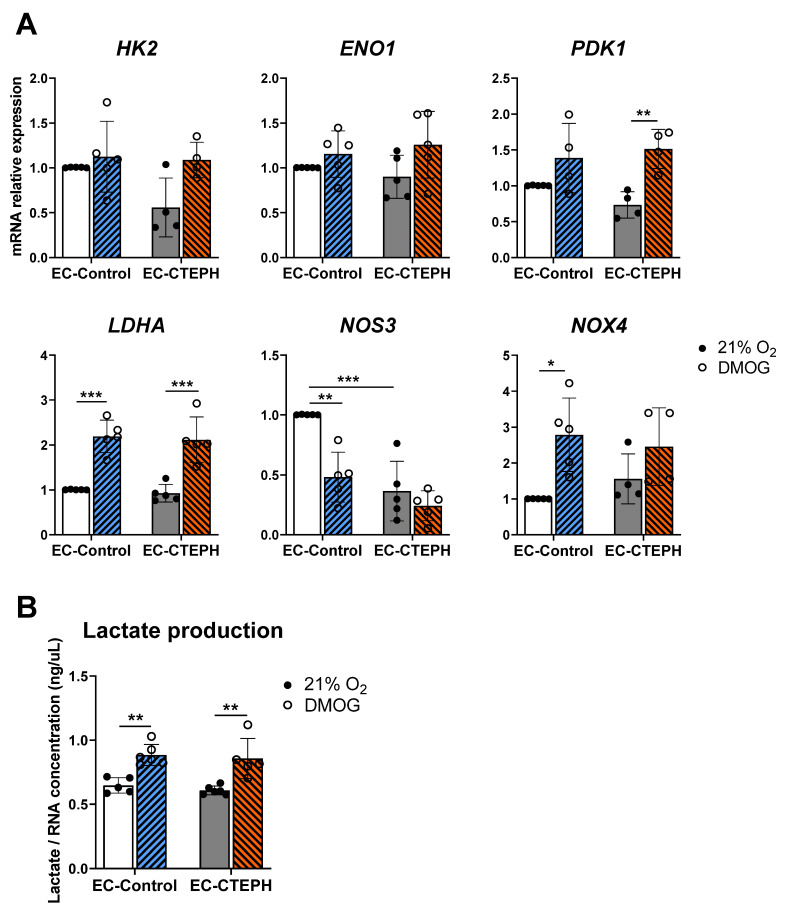
Expression of different subsets of genes in EC-Control and EC-CTEPH after dimethyloxalylglycine (DMOG) treatment. (**A**) mRNA relative expression of metabolic genes *HK2*, *ENO1*, *PDK1*, *LDHA*, angiogenic gene *NOS3*; and oxidative stress gene *NOX4*, in five independent experiments with *n* = 3 EC-Control at different passages and *n* = 5 EC-CTEPH. Statistical significance was indicated as *p* < 0.05 *, *p* < 0.01 **, *p* < 0.001 *** (2-way ANOVA and Tukey’s post hoc test). Each data point represents the mean ± SD of the relative fold change with respect to EC-Control normoxia basal levels (21% O_2_) normalized to reference gene *ACTB*. (**B**) Lactate released in cellular media normalized by RNA concentration in ng/µL in 5 independent experiments with *n* = 3 EC-Control at different passages and *n* = 5 EC-CTEPH, both in DMOG treatment and normoxia, *p* < 0.01 **, 2-way ANOVA and Tukey’s post hoc test. Values expressed as mean ± SD.

Chemical activation of the HIF pathway via PHD inhibition with dimethyloxalylglycine (DMOG) led to a comparable upregulation of *LDHA* in both EC-Control and EC-CTEPH cells compared to normoxia (0.92 ± 0.19 vs. 2.12 ± 0.51; and 1.01 ± 0.01 vs. 2.19 ± 0.36, *p* < 0.001, respectively) (Figure 9A), consistent with its induction under hypoxia (Figure 5A). *PDK1* expression also increased in both cell types upon DMOG treatment, with a more pronounced and statistically significant induction in EC-CTEPH (1.51 ± 0.27 vs. 0.74 ± 0.18, *p* < 0.01) (Figure 9A). The other genes, such as *HK2*, *ENO1* and *NOX4*, also showed a tendency to increase but did not reach statistical significance (Figure 9A).

Both *LDHA* mRNA expression and lactate accumulation in the culture medium showed significant differences across conditions, in line with previous results (Figure 9B).

No significant changes were observed in the expression of angiogenesis-related genes, including *PFK1*, *VEGFA*, *VWF*, *ICAM1*, *FGF2*, and *CD31*, in response to DMOG in either EC-Control or EC-CTEPH cells (Supplementary Figure S3). Also, NOS3 levels were significantly lower in EC-Control following DMOG administration (Figure 9A).

### 2.9. EC-CTEPH Results in Greater Oxidative Stress at Basal Levels than Does EC-Control

**Figure 10:**
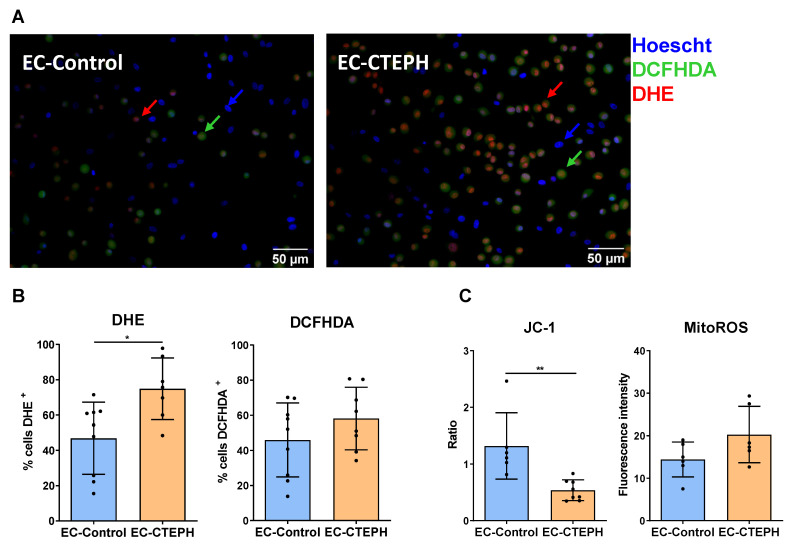
Basal oxidative stress and mitochondrial function. (**A**) Representative fluorescence microscopy images using 2′,7′-Dichlorofluorescin diacetate (DCFHDA, green), Dihydroethidium (DHE, red), and Hoechst (blue) under normoxia. 20× images; scale bar = 50 µm. Images were acquired individually and merged using ImageJ. (**B**) Quantification of percentage of DCFHDA^+^ and DHE^+^ cells. Data include four independent experiments for EC-Control (*n* = 3) and EC-CTEPH (*n* = 3) at passages 4–6, with two technical replicates and ten images per replicate. Values are mean ± SD. Statistical significance: *p* < 0.05 * (Mann–Whitney U test). (**C**) Mitochondrial membrane potential (JC-1) and mitochondrial ROS levels (MitoROS) in EC-Control and EC-CTEPH. Measurements were corroborated by microscopy. Data include three independent experiments with two technical replicates per cell line. Values are mean ± SD; *p* < 0.01 **, Mann–Whitney U test.

Under normoxic conditions, EC-CTEPH cells showed significantly higher DHE fluorescence compared with EC-Control (74.85 ± 17.48 vs. 46.86 ± 20.44, *p* < 0.05), indicating increased superoxide production (Figure 10A,B). The proportion of DCFHDA^+^ cells, reflecting overall ROS levels, did not reach statistical significance between groups.

JC-1 analysis revealed a trend toward mitochondrial membrane depolarization in EC-CTEPH compared with EC-Control (Figure 10C), suggesting mitochondrial dysfunction associated with the elevated oxidative stress observed in these cells.

### 2.10. Limited Transcriptional Response to H_2_O_2_-Induced Oxidative Stress in EC-CTEPH

**Figure 11:**
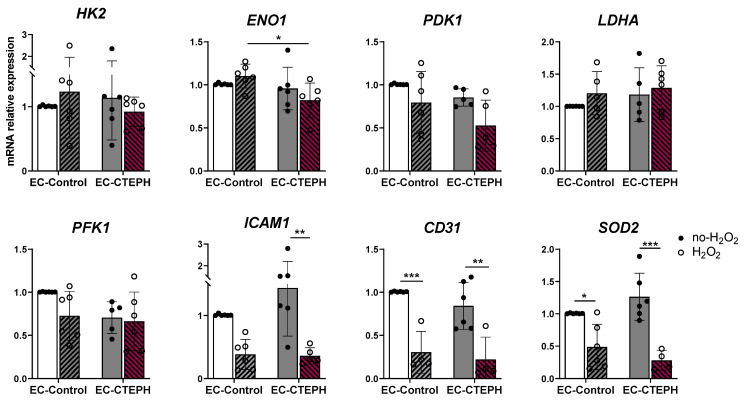
Expression of different subsets of genes in EC-Control and EC-CTEPH after hydrogen peroxide (H_2_O_2_) treatment. mRNA relative expression of metabolic genes *HK2*, *ENO1*, *PDK1*, *LDHA*, *PFK1*, angiogenic genes *ICAM1*, *CD31*; and oxidative stress gene *SOD2* in 6 independent experiments with *n* = 3 EC-Control at different passages and *n* = 5 EC-CTEPH at different passages. Statistical significance was indicated as *p* < 0.05 *, *p* < 0.01 **, *p* < 0.001 *** (2-way ANOVA and Tukey’s post hoc test). Each data point represents mean ± SD of the relative fold change with respect to EC-Control normoxia basal levels (21% O_2_) normalized to reference gene *ACTB*.

Under these conditions, H_2_O_2_ treatment did not significantly alter the expression of the tested metabolic genes (*HK2*, *ENO1*, *PDK1*, *LDHA*, *PFK1*) when comparing treated vs. untreated conditions within each group. However, the direction of the changes differed between EC-Control and EC-CTEPH cells (Figure 11). For instance, *ENO1* expression increased slightly in EC-Control cells after H_2_O_2_ exposure (1.10 ± 0.14 vs. 1.00 ± 0.01), whereas it decreased in EC-CTEPH cells (0.82 ± 0.19 vs. 1.00 ± 0.06), resulting in significantly lower *ENO1* levels in EC-CTEPH compared to EC-Control after treatment (*p* < 0.05) (Figure 11).

Regarding antioxidant and endothelial markers, *CD31* and *SOD2* were significantly downregulated in EC-Control cells after H_2_O_2_ treatment (*CD31*: 0.30 ± 0.24 vs. 1.00 ± 0.01, *p* < 0.001; *SOD2*: 0.49 ± 0.35 vs. 1.01 ± 0.01, *p* < 0.001). In EC-CTEPH cells, *CD31*, *ICAM1*, and *SOD2* expression also decreased significantly compared to their respective untreated conditions (*CD31*: 0.22 ± 0.26 vs. 0.84 ± 0.27, *p* < 0.01; *ICAM1*: 0.36 ± 0.13 vs. 1.44 ± 0.77, *p* < 0.01; *SOD2*: 0.28 ± 0.15 vs. 1.26 ± 0.37, *p* < 0.001) (Figure 11). No significant changes were observed in the expression of angiogenesis-related genes, including *VEGFA*, *VWF*, and *FGF2*, nor in oxidative stress gene *NOX4*, in response to H_2_O_2_ exposure in either EC-Control or EC-CTEPH cells (Supplementary Figure S4)

Overall, these findings show that H_2_O_2_ exposure induces changes in the expression of endothelial and antioxidant markers in both EC-Control and EC-CTEPH cells, while metabolic gene expression remains largely unaffected under the conditions tested.

## 3. Discussion

This study provides new insight into impaired endothelial adaptation to hypoxia in chronic thromboembolic pulmonary hypertension (CTEPH). Using a newly developed hypoxia-on-a-chip system, we show that pulmonary artery endothelial cells from CTEPH patients (EC-CTEPH) display a markedly attenuated transcriptional, metabolic, and functional response to low oxygen compared with healthy human pulmonary artery endothelial cells (EC-Control). These findings reveal intrinsically significant differences in endothelial behavior between EC-CTEPH and EC-Control, which may help explain the mechanisms underlying thrombus persistence and could inform future studies aimed at better understanding disease mechanisms and exploring potential translational implications.

The hypoxia-on-a-chip platform enabled precise temporal control of oxygen levels in the liquid phase, allowing cells to be exposed immediately and continuously to defined hypoxic conditions throughout the experiment. Using this approach, we were able to distinguish early and late hypoxic responses in EC-Control cells, whereas EC-CTEPH cells did not exhibit this temporal response pattern. Together, these findings underscore the importance of considering temporal dynamics when investigating endothelial adaptation to hypoxia.

Under hypoxia, both EC-Control and EC-CTEPH exhibited increased perimeter and nuclear area, consistent with stress-induced cell enlargement [22] and cytoskeletal rearrangement [23]. Proliferation was not significantly affected, in line with previous reports showing context-dependent effects of hypoxia on endothelial proliferation [24,25]. In contrast, EC-CTEPH showed delayed wound closure under hypoxia, particularly at 24 h, indicating impaired migration and repair capacity. This defect may contribute to reduced endothelial regeneration and persistent pulmonary hypertension after PEA [26].

*BNIP3* induction was observed only in EC-Control, suggesting partial activation of hypoxia-induced cell death pathways [27,28,29,30,31]. As *BNIP3* mediates a caspase-independent, necrosis-like cell death [27,32,33], its absence in EC-CTEPH supports a blunted stress response and maladaptive survival under hypoxia [7,25,34]. *TP53* and *PAK1* expression remained unchanged in both groups, indicating that senescence was not a major contributor under these experimental conditions. Further studies are needed to determine whether differential *BNIP3* expression affects cell death susceptibility.

Although angiogenesis was not directly assessed due to technical limitations of the hypoxia-on-a-chip platform, the lack of *VEGFA* and *NOS3* induction under hypoxic conditions in EC-CTEPH is consistent with impaired angiogenic signaling. Previous studies have linked defective angiogenesis to maladaptive vascular remodeling and persistent pulmonary hypertension in CTEPH [35,36,37]. Therefore, our findings reinforce the concept that impaired angiogenic signaling is a central mechanism in the pathophysiology of CTEPH, and may explain, at least in part, the limited capacity for vascular repair observed in these patients.

At the transcriptional level, EC-Control upregulated glycolytic (*HK2*, *ENO1*, *PDK1*, *LDHA*) and angiogenic (*VEGFA*, *NOS3*, *VWF*, *ICAM1*) genes under hypoxia, consistent with canonical HIF signaling [15,16,18]. In contrast, EC-CTEPH failed to activate these pathways despite HIF-1α and HIF-2α accumulation. This suggests a defect downstream of HIF stabilization that deserves further investigation. Possible mechanisms include epigenetic changes, altered chromatin accessibility, or impaired recruitment of transcriptional co-activators. Metabolic alterations influencing cofactor availability or redox balance may also contribute.

This blunted transcriptional response is consistent with previous reports of altered metabolism and reduced glycolytic activation in CTEPH endothelial cells [38,39,40,41]. It may also represent a context-dependent adaptation to chronic hypoxia. Future studies are needed to determine whether this phenotype is maladaptive or protective. Notably, EC-Control displayed distinct early (2–6 h) and late (48 h) hypoxic responses, whereas EC-CTEPH lacked this dynamic behavior, supporting defective adaptive signaling [25,42].

Reoxygenation following hypoxia restored gene expression in EC-Control, highlighting endothelial plasticity. In contrast, EC-CTEPH remained largely unresponsive. This may reflect persistent molecular alterations, such as chromatin remodeling or mitochondrial dysfunction [25,34,43,44], and suggests a stable hypoxia-associated phenotype. Whether this represents “hypoxic memory” requires further investigation.

Pharmacological HIF stabilization with dimethyloxalylglycine (DMOG) partially restored metabolic gene expression, indicating that impaired PHD–HIF signaling contributes to the observed phenotype [45,46,47]. However, expression of key genes such as *VEGFA*, *HK2* and *ENO1* was not restored, suggesting gene-specific regulation mediated by additional oxygen-sensitive factors [48,49,50,51,52,53].

Importantly, the partial response to DMOG indicates that the HIF transcriptional machinery remains at least partly functional in EC-CTEPH. This finding supports the concept that the altered response to physiological hypoxia may arise, at least in part, from defects in upstream oxygen-sensing mechanisms rather than from a complete disruption of HIF signaling.

Accordingly, pharmacological modulation of the PHD–HIF axis can partially modify the hypoxic response in EC-CTEPH, although these effects appear to be selective and incomplete. Further studies are required to clarify the underlying mechanism and to assess the potential therapeutic relevance of PHD inhibition in this context.

EC-CTEPH also showed marked mitochondrial dysfunction, including increased membrane depolarization and reduced integrity at baseline. These alterations were associated with elevated superoxide production [7,38] and a limited transcriptional response to exogenous H_2_O_2_, indicating an intrinsic redox imbalance [54]. The inability to induce antioxidant genes such as *NOX4* and *SOD2*, together with persistently low *NOS3* expression [7,38], suggests sustained oxidative stress. This may further impair endothelial signaling, metabolism, and nitric oxide-dependent regulation of inflammatory responses [55].

Together, mitochondrial dysfunction, redox imbalance, and impaired hypoxic adaptation may promote endothelial injury and maladaptive vascular remodeling in CTEPH [10,11,25,56]. These results suggest that exogenous oxidative stress elicits relatively limited and variable transcriptional responses under the conditions tested, and point to differential sensitivity between EC-Control and EC-CTEPH cells rather than a complete lack of responsiveness.

In summary, these findings reveal profound alterations in oxygen-sensing and adaptive pathways in EC-CTEPH, affecting hypoxia, reoxygenation, and redox homeostasis. These results provide a step forward in understanding intrinsic endothelial alterations that may underlie thrombus formation or persistence.

Importantly, these findings identify disease-relevant cellular features that may help to uncover fundamental mechanisms of CTEPH pathogenesis and support the development of essential novel curative therapies targeting the underlying endothelial dysfunction in CTEPH.

## 4. Materials and Methods

An expanded Materials and Methods section is available in the online document.

### 4.1. Ethics Statement

The study was conducted in accordance with the 1964 Declaration of Helsinki. The study protocol was granted by the Hospital Clínic of Barcelona ethics committee (HCB/2018/0837 and HCB/2018/0434), and all the subjects provided written informed consent.

### 4.2. Consent to Participate

All samples were collected with written informed consent from the participants.

### 4.3. Primary Cell Cultures

Five primary endothelial cell (EC) lines from different CTEPH patients were isolated from the luminal surface of proximal pulmonary arteries obtained from pulmonary endarterectomy (PEA) specimens collected from patients diagnosed with CTEPH at Hospital Clínic of Barcelona, Spain. Patient characteristics are summarized in Supplementary Table S2.

Endothelial isolation was performed following our previously published protocol [7]. During dissection, fibrotic and non-vascular areas were carefully excluded to ensure endothelial purity (Supplementary Figure S7). Briefly, fresh vascular tissue was minced and cultured in EGM-2 medium supplemented with 20% FBS (HyClone) until endothelial colonies appeared. The endothelial nature and purity of these cell lines had been thoroughly established in Tura-Ceide et al., 2021 [7,37]. The resulting cell lines were referred to as EC-CTEPH.

Three human pulmonary artery primary ECs isolated under similar conditions (Lonza) were used as controls, hereafter referred to as EC-Control. All experiments were conducted using EC-Control and EC-CTEPH between passages 4 and 6 to minimize phenotypic variability associated with cell passaging.

### 4.4. Design and Development of an In Vitro Hypoxia Model

The in vitro experimental system used in this study to induce hypoxia in cells was based on the method previously published [57]. Briefly, negative molds of two different chips were designed with the 123D Design program v2.2.14 and Ultimaker Cura software 5.11.0 (Ultimaker, Utrecht, Netherlands) and 3D-printed with an Ultimaker S5 3D printer (Ultimaker, Utrecht, Netherlands) in polycarbonate material. Chips consisted of cylindrical Polydimethylsiloxane (PDMS) wells with a 10 µm thin PDMS membrane. Inlet tubing was connected to a gas source that provided a constant mixture of gases. Gases flowed through the tubing and diffused homogeneously to all the wells.

The hypoxia-on-a-chip system reproduces key cellular responses observed in traditional hypoxia models, including HIF stabilization, upregulation of canonical glycolytic genes, and morphological adaptations in EC-Control cells. While a direct side-by-side comparison with conventional culture conditions was not performed in this study, these results align with previously published data using standard hypoxia systems [15,16,18].

### 4.5. Oxygen Measurements

Oxygen pressure (PO_2_) measurements (in mmHg) were taken with a fiber-optic oxygen sensor (FireSting O_2_, Pyro Science, Aachen, Germany) placed above the surface of the permeable membrane at the cell culture level, as well as in standard cell culture dishes. Prior to measurement, the sensor was calibrated following the manufacturer’s instructions via two reference points: air saturated with water (21% O_2_) and an anoxic solution of 0.1 M sodium ascorbate and sodium hydroxide. The measures were then transformed into percentages via the following formula:

$$\%O_{2}=\frac{O_{2}\;partial\;pressure\;(in\;mmHg)}{760}\times 100$$

### 4.6. Hypoxia Experimental Setup

EC-Control and EC-CTEPH were seeded in 6-well chips for migration, proliferation and morphology assessment and in 4-well chips for RNA or protein extraction. Hypoxia was induced for 48 h unless otherwise specified. For reoxygenation experiments, the cells were exposed to 48 h of hypoxia, followed by 48 h of 21% O_2_.

Both the EC-Control and EC-CTEPH samples were exposed to different O_2_ concentrations: 1%, 4%, 13% and 21% O_2_.

### 4.7. Cell Migration and Wound Healing Assays

EC-Control and EC-CTEPH were seeded in 6-well chips in complete media (EGM-2 media supplemented with 10% FBS (HyClone)). The media was replaced with starvation media, which consisted of EGM-2 media supplemented with 2% FBS and 2 mM hydroxyurea, 24 h prior to the assay. A straight line was made across each well with a pipette tip. Images of the wounds were taken at times t = 0 h, t = 6 h, t = 8 h, t = 10 h, t = 24 h, t = 30 h and t = 48 h with a Zeiss Axiovert 200 microscope (Oberkochen, Germany) at 10× magnification. Images were treated with the GIMP program and analyzed with ImageJ. Wound closure was expressed as the percentage of the wound healed divided by the area of the original wound at 0 h.

### 4.8. Cell Morphology

EC-Control and EC-CTEPH were seeded in 6-well chips in complete media until a confluent monolayer formed. After 48 h at the desired O_2_ concentration, 1% and 21% O_2_ pictures were taken at 20× magnification with a Zeiss Axiovert 200 microscope. The perimeter, cellular area and nuclear area were measured in a minimum of 10 representative cells per image via ImageJ software v.1.54k.

### 4.9. Cell Proliferation

Both cell phenotypes were seeded in 4-well chips in complete media (100,000 cells/well). After 48 h of hypoxia induction, the cells were trypsinized and counted with trypan blue in a Neubauer chamber. Four independent experiments were conducted.

### 4.10. Gene Expression Analysis

The mRNA levels of different subsets of genes were measured via RT-qPCR. ECs were seeded in 4-well chips in complete media and switched to starvation media 24 h prior to exposure to different O_2_ concentrations. Total RNA was extracted using TRIsure (Bioline, Luckenwalde, Germany) and quantified via spectrophotometry (Nanodrop 2000c, Thermo Fisher, Waltham, MA, USA). Reverse transcription was performed using a High-Capacity cDNA Reverse Transcription Kit (Applied Biosystems, Foster City, CA, USA).

SYBR Green I (Thermo Fisher Scientific, Waltham, MA, USA) and specific primers were used for RT-qPCR on a ViiA7 Real-Time PCR system (Applied Biosystems, Foster City, CA, USA). Relative quantification was performed by normalizing the Ct (threshold cycle) of the gene of interest to that of the endogenous control β-actin in the same sample and to normoxia for each condition using the comparative 2^−(ΔΔCt)^ method.

The analyzed genes included key glycolytic enzymes (*HK2*, *ENO1*, *PDK1*, *LDHA*), angiogenesis-related genes (*VEGFA*, *NOS3*, *VWF*, *ICAM1*, *FGF2*, *CD31*), oxidative stress-related genes (*NOX4*, *SOD2*), apoptosis-related genes (*BNIP3*, *CASP8*, *CASP9*, *PAK1*, *TP53*), and genes involved in the regulation of the HIF signaling pathway and cellular hypoxia responses (*EGLN1*, *EGLN3*, *HIF1AN*). All primers were designed with Primer3 Plus, v.3.2.0, and synthesized by Integrated DNA Technologies. Primer sequences are shown in Supplementary Table S3.

### 4.11. Lactate Production

EC-Control and EC-CTEPH were seeded in 4-well chips in complete media. The media was then replaced with starved media 24 h prior to exposure to different O_2_ concentrations for 48 h. The cellular supernatant was collected and measured with Epoc BGEM Blood Test cards (Siemens, Munich, Germany). The lactate values obtained were normalized to the total RNA for each sample.

### 4.12. Protein Expression Analysis

Proteins were analyzed via two different protocols. First, cells cultured in vitro were homogenized and denatured directly in Laemmli buffer, loaded onto SDS-polyacrylamide gels and transferred onto nitrocellulose membranes (Cytiva, Marlborough, MA, USA). Second, cells cultured in vitro were treated with ice-cold Pierce RIPA buffer (Thermo Fisher-Scientific, Waltham, MA, USA) supplemented with a Halt protease/phosphatase inhibitor cocktail (Thermo Fisher-Scientific, USA). After incubation on ice, the cell lysate was recovered and centrifuged, and the protein concentration was determined via a Pierce BCA protein assay kit (Thermo Fisher-Scientific, USA). The samples were loaded onto 4–12% Bis-Tris gels (Thermo Fisher-Scientific, USA), and the proteins were transferred onto nitrocellulose membranes. Following blocking, the membranes were probed with the antibodies shown in Supplementary Table S4. Immunoreactivity was detected through enhanced chemiluminescence, utilizing Clarity Western ECL Substrate (Bio-Rad, Hercules, CA, USA), SuperSignal West Femto Maximum Sensitivity Substrate (Thermo Fisher-Scientific, USA), or WesternBright Quantum substrate (Advansta, San Jose, CA, USA) and visualized on an Image Quant LAS4000 Mini (GE Healthcare, Chicago, IL, USA). Densitometric analysis of the images was performed via ImageLab 6.1 software. Data were obtained from four independent experiments (*n* = 3 EC-Control and *n* = 4 EC-CTEPH, passages 4–6). Full-length gels are shown in Supplementary Figures S8 and S9.

### 4.13. Chemical Induction of Hypoxia by Dimethyloxalylglycine (DMOG) Administration

Both EC-Control and EC-CTEPH cells were cultured in complete medium until confluence. The medium was then replaced with complete medium containing 1 mM dimethyloxalylglycine (DMOG; MedChem, Princeton, NJ, USA) for 24 h. DMOG is a prolyl hydroxylase (PHD) inhibitor that stabilizes hypoxia-inducible factors (HIFs), thereby mimicking hypoxia under normoxic conditions. This approach was used in vitro to determine whether the impaired hypoxic response in EC-CTEPH cells was due to defective HIF stabilization or to downstream transcriptional dysfunction. Gene expression and lactate production were analyzed as described in Section 4.10 and Section 4.11, respectively.

### 4.14. Radical Oxidative Species Measurements

Basal reactive oxygen species (ROS) levels were measured in EC-Control and EC-CTEPH cells using specific dyes. Intracellular hydrogen peroxide (H_2_O_2_) was assessed with 2′,7′-Dichlorofluorescin diacetate (DCFHDA, 50 µM), and superoxide (O_2_^−^) with dihydroethidium (DHE, 50 µM), both in phenol-red-free EGM-2 media supplemented with 2% FBS. Hoechst (30 nM) was used to normalize for cell number. Cells were incubated overnight, and fluorescence was read with a Synergy HTX Multi-Mode microplate reader (DHE: Ex/Em 518/606 nm; DCFHDA: Ex/Em 495/520 nm; Hoechst: Ex/Em 350/461 nm). Additionally, fluorescence images were acquired with a Zeiss Axiovert 200 microscope at 20× magnification and merged using ImageJ. Data were collected from four independent experiments, each including *n* = 3 EC-Control and *n* = 3 EC-CTEPH cell lines (passages 4–6), with two technical replicates and ten images per replicate.

### 4.15. Mitochondrial Oxidative Stress

To assess mitochondrial reactive oxygen species (ROS) production, the MitoSOX™ Mitochondrial Superoxide Indicator (Cayman, Ann Arbor, MI, USA) was utilized. A stock solution of 5 mM MitoSOX was diluted in HBSS containing calcium and magnesium (HBSS/Ca/Mg) to prepare a 5 μM working solution. This was applied to cells cultured to approximately 80% confluence in 0.2% gelatin-coated 24-well plates. Cells were incubated with the reagent for 20 min at 37 °C, then washed three times with HBSS/Ca/Mg. Following this, an additional incubation period of 1 h at 37 °C was performed. Fluorescence was recorded using a Synergy HTX BioTek microplate reader (Agilent, Santa Clara, CA, USA) at an excitation wavelength of 480 nm and emission at 560 nm. To corroborate the plate reader data, fluorescence signals were also visualized and confirmed via microscopy using a Nikon Eclipse 50i (Tokyo, Japan).

### 4.16. Mitochondrial Membrane Potential

The mitochondrial membrane potential was analyzed using the JC-1 Mitochondrial Membrane Potential Assay Kit (ThermoFisher Scientific, USA). A 200 μM JC-1 stock solution was diluted in EGM-2 medium supplemented with 10% FBS and 1% penicillin/streptomycin to obtain a 2 μM working solution. This solution was added to cells seeded in 0.2% gelatin-coated 24-well plates at 80% confluence. Following a 30 min incubation at 37 °C, cells were rinsed twice with PBS. Fluorescence was measured using a Synergy HTX BioTek spectrophotometer (Agilent, USA), with detection of JC-1 red aggregates at 550 nm excitation and 600 nm emission and JC-1 green monomers at 485 nm excitation and 535 nm emission (Molecular Probes, Eugene, OR, USA, 2002). A decrease in the red-to-green fluorescence intensity ratio indicated a loss of mitochondrial membrane potential in endothelial cells. Microscopy analysis (Nikon Eclipse 50i) was performed in parallel to validate the plate reader measurements.

### 4.17. Oxidative Stress Induction by Hydrogen Peroxide (H_2_O_2_) Administration

Both EC-Control and EC-CTEPH were cultured in complete media until they reached confluence in 4-well chips. The media was replaced with a solution containing starvation media supplemented with 0.5 mM H_2_O_2_ for 1 h. Gene expression analysis was performed as described in Section 4.10 of the Materials and Methods section. The protocol was adapted from [58]. 

### 4.18. Statistical Analyses

Statistical analyses were performed using GraphPad Prism 7 software (version 7.0e). Data are presented as mean ± SD. Normality was assessed using the Shapiro–Wilk test. As several datasets did not follow a normal distribution, non-parametric tests were applied as a conservative approach when appropriate.

Independent samples were analyzed using the Mann–Whitney U test. For comparisons across different O_2_ conditions, one-way ANOVA with Dunnett’s post hoc test or two-way ANOVA with Tukey’s post hoc test were used, as appropriate. Statistical significance was defined as *p* ≤ 0.05 (α = 0.05).

Each experiment was performed using independent primary cell lines derived from different donors, with technical replicates included for each condition.

## 5. Conclusions

In summary, EC-CTEPH show an attenuated molecular and functional response to hypoxia and reoxygenation, with altered oxidative stress homeostasis and impaired HIF-dependent transcriptional activation. These alterations may contribute to sustained endothelial dysfunction in CTEPH. Pharmacological HIF stabilization partially restores gene expression profiles, providing proof-of-concept for further exploration of targeted therapeutic strategies. These findings have future translational relevance: they may provide a conceptual framework for future studies investigating potential biomarkers, disease mechanisms, and therapeutic applications in CTEPH.

## 6. Limitations

Certain limitations should be acknowledged in this study.

First, EC-CTEPH cells were obtained exclusively from patients who underwent PEA and therefore represent advanced operable CTEPH; endothelial cells from inoperable or distal disease phenotypes may exhibit different responses. In addition, the relatively small sample size and limited number of primary endothelial cell lines, although consistent with comparable published in vitro studies, may restrict generalizability, increase the influence of inter-individual biological variability, and limit statistical power to detect subtle effects. Moreover, the limited replicative capacity of primary cells highlights the need for validation in larger cohorts and, although this is currently very challenging, ideally in vivo models.

Metabolic adaptation was inferred from gene expression analyses and lactate measurements rather than direct metabolic flux analysis. Future studies incorporating ECAR/OCR measurements would help further define metabolic alterations in EC-CTEPH.

Apoptosis was assessed primarily at the mRNA level for selected genes, providing an initial indication of pathway activation without protein-level or functional validation; complementary assays such as TUNEL or Annexin V/PI staining will be required to confirm these observations. This limitation was largely related to the culture of cells on a thin PDMS membrane within the chip rather than on a conventional rigid surface. Although this configuration was well-suited for gas exchange and hypoxia induction, it posed technical challenges for certain assays, including immunofluorescence and Matrigel-based tube formation or sprouting. Consequently, angiogenic capacity was inferred mainly from gene expression changes rather than direct functional angiogenesis assays. Future work should focus on refining these methods and incorporating complementary assays to strengthen the functional validation of our findings.

## Data Availability

All data relevant to the study are included in the article or uploaded as online information.

## Supplementary Materials

The following supporting information can be downloaded at: https://www.mdpi.com/article/10.3390/ijms27073207/s1.

## Abbreviations

The following abbreviations are used in this manuscript:

*ACTB*	actin beta gene
BMI	body mass index
*BNIP3*	BCL2 interacting protein 3 gene
BNP	B-type natriuretic peptide
*CASP8*	Caspase 8 gene
*CASP9*	Caspase 9 gene
*CD31*	platelet endothelial cell adhesion molecule gene
CTEPH	chronic thromboembolic pulmonary hypertension
DCFHDA	2′,7′-Dichlorofluorescin diacetate
DHE	dihydroethidium
DMOG	dimethyloxalylglycine
ECs	endothelial cells
ED	endothelial dysfunction
EGM-2	endothelial growth media 2
*EGLN1*	prolyl hydroxylase domain-containing protein 2 gene
*EGLN3*	prolyl hydroxylase domain-containing protein 3 gene
*ENO1*	alpha enolase gene
NOS3	endothelial nitric oxide synthase protein
ET-1	endotelin-1
FC	functional class
*FGF2*	fibroblast growth factor 2 gene
HIF1AN	hypoxia-inducible factor 1 alpha subunit inhibitor protein
H_2_O_2_	hydrogen peroxide
HIF	hypoxia-inducible factor
*HIF-1*	hypoxia-inducible factor 1 gene
*HIF-2*	hypoxia-inducible factor 2 gene
HIF-1α	hypoxia-inducible factor 1, alpha subunit protein
HIF-2α	hypoxia-inducible factor 2, alpha subunit protein
*HIF1AN*	hypoxia-inducible factor 1 alpha subunit inhibitor gene
*HK2*	hexokinase 2 gene
*ICAM1*	intercellular adhesion molecule 1 gene
*LDHA*	lactate dehydrogenase A gene
LDHA	lactate dehydrogenase A protein
mmHg	millimeters of mercury
mPAP	mean pulmonary artery pressure
NO	nitric oxide
*NOS3*	endothelial nitric oxide synthase gene
*NOX4*	NADPH oxidase 4 gene
O_2_	oxygen
O_2_^−^	superoxide radical
PAH	pulmonary arterial hypertension
*PAK1*	cyclin-dependent kinase inhibitor 1A gene
*PDK1*	pyruvate dehydrogenase kinase 1 gene
PDK1	pyruvate dehydrogenase kinase 1 protein
PDMS	polydimethylsiloxane
PEs	pulmonary emboli
PEA	pulmonary endarterectomy
*PFK1*	phosphofructokinase 1 gene
PH	pulmonary hypertension
PHD	prolyl hydroxylase domain
PO_2_	oxygen pressure
PVD	pulmonary vascular disease
PVR	pulmonary vascular resistance
ROS	radical oxygen species
*SOD2*	superoxide dismutase 2 gene
SvO_2_	mixed venous oxygen saturation
*TP53*	tumor protein 53 gene
*VEGFA*	vascular endothelial growth factor gene
*VWF*	von Willebrand factor gene
WHO	World Health Organization
