# Supplementary material for: Understanding the Impact of Hypoxia on Pulmonary Artery Endothelial Cells in Chronic Thromboembolic Pulmonary Hypertension Patients
Source: Int J Mol Sci. 2026 Apr 1;27(7):3207. doi: 10.3390/ijms27073207 (PMC13074002; doi:10.3390/ijms27073207)
Supplement: Supplementary file 1 [file ijms-27-03207-s001.zip › ijms-4175807-supplementary.pdf]

Supplementary Material.

UNDERSTANDING THE IMPACT OF HYPOXIA ON PULMONARY ARTERY ENDOTHELIAL CELLS IN CHRONIC THROMBOEMBOLIC PULMONARY HYPERTENSION PATIENTS

Ylenia Roger[1,2], Anna Sardiné-Rama[1,2], Adelaida Bosacoma[1,2], Irene Gómez[1], Rita Fernández-Hernández[1], Francisco Rafael Jimenez-Trinidad[3], Cristina Rodríguez[1], Cristina Bonjoch[1], Isaac Almendros[1,2,4], Esther Marhuenda[4], Andrés Amalio Urrutia[5], Míriam Peracaula[6], Manuel Castellà[7], Isabel Blanco[1,2], Ana Ramírez[1], Víctor Ivo Peinado[1,2,8], Joan Albert Barberà[1,2,+] Olga Tura-Ceide[1,2,9,+]

[+]**Corresponding authors**: olgaturac@gmail.com, ORCID number: 000-0003-4334-9790 and jbarbera@clinic.cat, ORCID number: 0000-0003-1469-4990. Servei de Pneumologia, Hospital Clínic Villarroel, 170. Barcelona 08036. Spain. Phone 34-93-227-5747; Fax. 34-93-227-5455.

DETAILED MATERIALS AND METHODS

Subjects

Five subjects with CTEPH, aged between 59-81 years who underwent PEA at the Hospital Clinic of Barcelona, Spain were enrolled in the study. Patient and Control characteristics are shown in Table S1. CTEPH was diagnosed according to current guidelines[1]. The study was conducted in accordance with the Declaration of Helsinki, approved by the institutional Committee on Human Research (Hospital Clínic of Barcelona ethics committee HCB/2018/0837 and HCB/2018/0434) and all subjects gave written informed consent.

Primary cell cultures

Isolated ECs (EC-CTEPH) from all n=5 subjects were obtained from fresh PEA resected specimens (Supplementary Figure 1) by mincing it into 1-2 mm pieces and culturing in 0.2% gelatin-coated plates in EBM-2 growth medium (Lonza), supplemented with 10% fetal bovine serum (FBS, Hyclone) and EGM-2 SingleQuots (Lonza). Cell colonies appeared after 7-20 days in culture. Cells were used between passages 4-6. All cellular experiments were performed at a cell confluency of 80-90% unless otherwise stated. Human pulmonary artery ECs (HPAE, EC-Control) n=3 were purchased from Lonza and used as control. Control lines were used at passages 4-6 and maintained in a humidified atmosphere at 37ºC in 5% CO_2.

Wound healing assay

Cell migration was evaluated using a scratch wound assay. Twenty thousand subconfluent EC-CTEPH and EC-Control were seeded in 6-well chips and starved (EGM-2 + 2% FBS + 2mM hydroxyurea) prior to scratching the cell monolayer with a p200 pipette tip to generate a wound. Non-adherent cells were removed by washing with PBS and normal growth medium was added for 48h. Pictures were taken at baseline, 6h, 8h, 10h, 24h, 30h and 48h. Wound closure was expressed as percentage of regrowth divided by area and width of original wound. The healing

area was analyzed with freely available imaging processing ImageJ software. Statistical analysis shown in Supplementary Table S5.

Cell morphology

Cellular circumference, cellular area and nuclear area were measured using freely available imaging processing ImageJ software, in triplicates fields of 10 cells/picture (20x magnification).

RNA Isolation and quantitative Real Time PCR

Total RNA was extracted from 80-90% confluent cultures using 1ml of TRIsure reagent (Bioline) according to the manufacturer instructions. Following reverse transcription (high-capacity cDNA RT kit, Applied Biosystems), quantitative real-time PCR experiments were performed in the presence of fluorescent dye (power SYBR Green, Applied Biosystems) with a ViiA 7 Real-Time PCR System (Applied Biosystems). cDNA copy numbers were normalized against genomic DNA level of endogenous β-actin and analyzed by the 2-ΔΔCt method. All primers were delivered by IDT and primer sequences are listed in Supplementary Table S2.

Western Blotting

Antibodies used for immunodetection are detailed in Supplementary Table S3. The intensity of the individual bands was quantified using freely available Image Lab software (Bio-Rad Laboratories). All results are shown as relative expression to β-actin or Vinculin protein levels.

SUPPLEMENTARY FIGURES

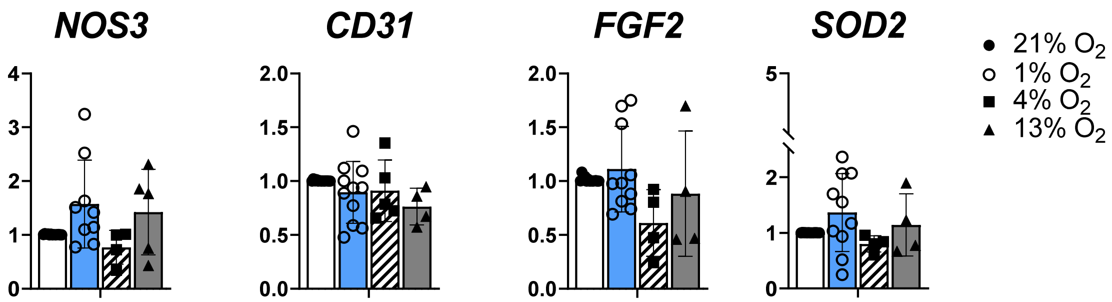

Supplementary Figure 1. Expression of different subsets of genes in EC-Control under different O_2 conditions. mRNA relative expression of angiogenic genes *NOS3*, *CD31*, *FGF2*; and oxidative stress gene *SOD2*, in 6 independent experiments with n=3 EC-Control at different passages. Each data point represented the mean ± SD of the relative fold change with respect to EC-Control normoxia basal levels (21% O_2) normalized to reference gene ACTB (One-way ANOVA and Dunnett's post-hoc test).

	EC-Control			EC-CTEPH		
VEGFA	2h	δ = 6,077<br>adjusted p-value = 0,0022	**	2h	δ = 3,572<br>adjusted p-value = 0,0022	**
	4h	δ = 2,985<br>adjusted p-value = 0,0022	**	4h	δ = 2,818<br>adjusted p-value = 0,0022	**
	6h	δ = 2,382<br>adjusted p-value <0,0001	****	6h	δ = 1,871<br>adjusted p-value = 0,0022	**
	12h	δ = 1,016<br>adjusted p-value = 0,431	ns	12h	δ = 1,261<br>adjusted p-value = 0,431	ns
	24h	δ = 1,723<br>adjusted p-value = 0,431	ns	24h	δ = 0,270<br>adjusted p-value = 0,0048	**
	48h	δ = 2,405<br>adjusted p-value = 0,0043	**	48h	δ = 6,146<br>adjusted p-value = 0,0022	**
CD31	2h	δ = 2,091<br>adjusted p-value = 0,0022	**	2h	δ = 0,862<br>adjusted p-value = 0,351	ns
	4h	δ = 1,305<br>adjusted p-value = 0,0022	**	4h	δ = 1,276<br>adjusted p-value = 0,0022	**
	6h	δ = 1,287<br>adjusted p-value > 0,999	ns	6h	δ = 1,008<br>adjusted p-value = 0,431	ns
	12h	δ = 0,768<br>adjusted p-value = 0,0022	**	12h	δ = 1,089<br>adjusted p-value = 0,431	ns
	24h	δ = 1,617<br>adjusted p-value = 0,0022	**	24h	δ = 0,964<br>adjusted p-value = 0,431	ns
	48h	δ = 1,012<br>adjusted p-value > 0,999	ns	48h	δ = 1,113<br>adjusted p-value = 0,431	ns
HK2	2h	δ = 5,935<br>adjusted p-value = 0,0022	**	2h	δ = 2,142<br>adjusted p-value = 0,0022	**
	4h	δ = 2,534<br>adjusted p-value = 0,0022	**	4h	δ = 3,170<br>adjusted p-value = 0,0022	**
	6h	δ = 2,145<br>adjusted p-value = 0,0022	**	6h	δ = 2,303<br>adjusted p-value = 0,0022	**
	12h	δ = 0,923<br>adjusted p-value = 0,431	ns	12h	δ = 1,001<br>adjusted p-value = 0,351	ns
	24h	δ = 4,330<br>adjusted p-value = 0,431	ns	24h	δ = 1,180<br>adjusted p-value = 0,431	ns
	48h	δ = 1,593<br>adjusted p-value = 0,0022	**	48h	δ = 4,271<br>adjusted p-value = 0,0022	**

PDK1	2h	δ = 3,777	**	2h	δ = 1,696	**
		adjusted p-value = 0,0022			adjusted p-value = 0,0022	
	4h	δ = 1,866	**	4h	δ = 2,191	**
		adjusted p-value = 0,0022			adjusted p-value = 0,0022	
	6h	δ = 1,674	**	6h	δ = 1,468	**
		adjusted p-value = 0,0022			adjusted p-value = 0,0022	
	12h	δ = 0,837	ns	12h	δ = 1,072	ns
		adjusted p-value = 0,351			adjusted p-value = 0,351	
	24h	δ = 1,676	**	24h	δ = 1,608	ns
		adjusted p-value = 0,0022			adjusted p-value = 0,431	
	48h	δ = 1,336	ns	6h	δ = 4,275	**
		adjusted p-value = 0,431			adjusted p-value = 0,0022	
FGF2	2h	δ = 1,311	**	2h	δ = 0,598	**
		adjusted p-value = 0,0022			adjusted p-value = 0,0022	
	4h	δ = 0,996	ns	4h	δ = 0,941	ns
		adjusted p-value = 0,368			adjusted p-value = 0,431	
	6h	δ = 0,828	ns	6h	δ = 0,971	ns
		adjusted p-value = 0,431			adjusted p-value = 0,431	
	12h	δ = 0,762	**	12h	δ = 0,997	ns
		adjusted p-value = 0,0022			adjusted p-value = 0,431	
	24h	δ = 1,438	**	24h	δ = 0,904	ns
		adjusted p-value = 0,0022			adjusted p-value = 0,431	
	48h	δ = 0,757	ns	48h	δ = 0,869	ns
		adjusted p-value = 0,431			adjusted p-value = 0,431	
NOS3	2h	δ = 0,900	ns	2h	δ = 0,737	ns
		adjusted p-value = 0,351			adjusted p-value = 0,431	
	4h	δ = 0,739	ns	4h	δ = 1,071	ns
		adjusted p-value = 0,351			adjusted p-value = 0,368	
	6h	δ = 0,985	ns	6h	δ = 0,930	ns
		adjusted p-value = 0,431			adjusted p-value = 0,870	
	12h	δ = 0,894	**	12h	δ = 1,077	ns
		adjusted p-value = 0,0022			adjusted p-value = 0,431	
	24h	δ = 1,071	ns	24h	δ = 0,990	ns
		adjusted p-value = 0,431			adjusted p-value = 0,351	
	48h	δ = 0,621	**	48h	δ = 0,522	**
		adjusted p-value = 0,0022			adjusted p-value = 0,0022	

LDHA	2h	δ = 2,111<br>adjusted p-value = 0,0022	**	2h	δ = 1,238<br>adjusted p-value = 0,0022	**
	4h	δ = 1,558<br>adjusted p-value = 0,0022	**	4h	δ = 1,919<br>adjusted p-value = 0,0022	**
	6h	δ = 1,866<br>adjusted p-value = 0,396	ns	6h	δ = 1,436<br>adjusted p-value = 0,0022	**
	12h	δ = 0,827<br>adjusted p-value = 0,431	ns	12h	δ = 1,255<br>adjusted p-value = 0,351	ns
	24h	δ = 1,888<br>adjusted p-value = 0,095	**	24h	δ = 1,150<br>adjusted p-value = 0,431	ns
	48h	δ = 1,947<br>adjusted p-value = 0,0022	**	48h	δ = 1,977<br>adjusted p-value = 0,0022	**
SOD2	2h	δ = 1,557<br>adjusted p-value = 0,0022	**	2h	δ = 0,861<br>adjusted p-value = 0,431	ns
	4h	δ = 1,301<br>adjusted p-value = 0,0022	**	4h	δ = 1,195<br>adjusted p-value = 0,0022	**
	6h	δ = 1,656<br>adjusted p-value = 0,0022	**	6h	δ = 0,942<br>adjusted p-value = 0,368	ns
	12h	δ = 0,946<br>adjusted p-value = 0,431	ns	12h	δ = 1,108<br>adjusted p-value = 0,431	ns
	24h	δ = 1,041<br>adjusted p-value = 0,431	ns	24h	δ = 0,950<br>adjusted p-value = 0,351	ns
	48h	δ = 0,874<br>adjusted p-value = 0,368	ns	48h	δ = 0,987<br>adjusted p-value = 0,368	ns
PFK1	2h	δ = 0,972<br>adjusted p-value = 0,431	ns	2h	δ = 0,778<br>adjusted p-value = 0,0022	**
	4h	δ = 0,890<br>adjusted p-value = 0,431	ns	4h	δ = 0,941<br>adjusted p-value = 0,431	ns
	6h	δ = 0,946<br>adjusted p-value = 0,351	ns	6h	δ = 0,638<br>adjusted p-value = 0,0022	**
	12h	δ = 0,959<br>adjusted p-value = 0,351	ns	12h	δ = 1,170<br>adjusted p-value = 0,431	ns
	24h	δ = 1,755<br>adjusted p-value = 0,368	ns	24h	δ = 8,234<br>adjusted p-value = 0,0095	**
	48h	δ = 0,777<br>adjusted p-value = 0,351	ns	48h	δ = 3,691<br>adjusted p-value = 0,431	ns
ENO1	2h	δ = 1,171<br>adjusted p-value = 0,030	*	2h	δ = 0,968<br>adjusted p-value = 0,431	ns
	4h	δ = 1,215<br>adjusted p-value = 0,0022	**	4h	δ = 1,99<br>adjusted p-value = 0,431	ns
	6h	δ = 1,521<br>adjusted p-value = 0,431	ns	6h	δ = 1,069<br>adjusted p-value = 0,431	ns
	12h	δ = 1,101<br>adjusted p-value = 0,431	ns	12h	δ = 0,966<br>adjusted p-value = 0,351	ns
	24h	δ = 1,995<br>adjusted p-value = 0,0022	**	24h	δ = 0,852<br>adjusted p-value = 0,431	ns
	48h	δ = 1,459<br>adjusted p-value = 0,0022	**	48h	δ = 3,350<br>adjusted p-value = 0,0022	**

NOX4	2h	δ = 1,015	adjusted p-value = 0,351	ns	2h	δ = 0,898	adjusted p-value = 0,368	ns
	4h	δ = 1,451	adjusted p-value = 0,368	ns	4h	δ = 1,151	adjusted p-value = 0,0022	**
	6h	δ = 2,460	adjusted p-value = 0,0022	**	6h	δ = 1,697	adjusted p-value = 0,368	ns
	12h	δ = 0,970	adjusted p-value = 0,431	ns	12h	δ = 1,016	adjusted p-value = 0,431	ns
	24h	δ = 1,278	adjusted p-value = 0,431	ns	24h	δ = 0,914	adjusted p-value = 0,351	ns
	48h	δ = 2,124	adjusted p-value = 0,0095	**	48h	δ = 1,346	adjusted p-value = 0,0022	**
ICAM1	2h	δ = 1,397	adjusted p-value = 0,431	ns	2h	δ = 0,736	adjusted p-value = 0,0022	**
	4h	δ = 1,902	adjusted p-value = 0,0022	**	4h	δ = 0,707	adjusted p-value = 0,431	ns
	6h	δ = 1,299	adjusted p-value = 0,431	ns	6h	δ = 1,014	adjusted p-value = 0,431	ns
	12h	δ = 0,876	adjusted p-value = 0,431	ns	12h	δ = 1,438	adjusted p-value = 0,431	ns
	24h	δ = 1,643	adjusted p-value = 0,0022	**	24h	δ = 0,969	adjusted p-value = 0,431	ns
	48h	δ = 1,331	adjusted p-value = 0,431	ns	48h	δ = 1,645	adjusted p-value = 0,368	ns
VWF	2h	δ = 1,732	adjusted p-value = 0,431	ns	2h	δ = 0,562	adjusted p-value = 0,0022	**
	4h	δ = 2,393	adjusted p-value = 0,431	ns	4h	δ = 0,597	adjusted p-value = 0,0022	**
	6h	δ = 1,747	adjusted p-value = 0,0022	**	6h	δ = 0,724	adjusted p-value = 0,0022	**
	12h	δ = 0,892	adjusted p-value = 0,431	ns	12h	δ = 1,238	adjusted p-value = 0,0022	**
	24h	δ = 1,734	adjusted p-value = 0,0022	**	24h	δ = 0,930	adjusted p-value = 0,351	ns
	4h	δ = 1,262	adjusted p-value = 0,084	ns	48h	δ = 1,099	adjusted p-value = 0,431	ns

Supplementary Table S1. Statistical analysis comparing hypoxia and normoxia conditions in EC-Control and EC-CTEPH cells, shown per gene and per time point in the heatmap. δ value represents the hypoxia/normoxia ratio. Statistical significance was indicated as $p<0.05$*, $p<0.01$**, $p<0.0001$**** (Mann-Whitney U test). Data were obtained from three independent experiments (n = 3 EC-Control and n = 3 EC-CTEPH, passages 4–6).

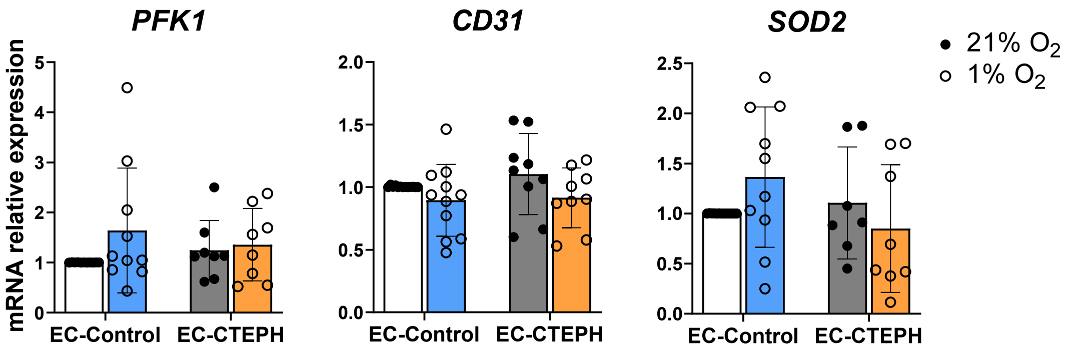

Supplementary Figure 2. Expression of different subsets of genes in EC-Control and EC-CTEPH under hypoxia. mRNA relative expression of metabolic gene *PFK1*, angiogenic gene *CD31,* and oxidative stress gene *SOD2*, in six independent experiments (n=3 EC-Control and n=5 EC-CTEPH, passages 4-6). Each data point represents the mean ± SD of the relative fold change compared to EC-Control normoxic basal levels (21% O_2), normalized to reference gene ACTB (2-way ANOVA followed by Tukey's post-hoc test).

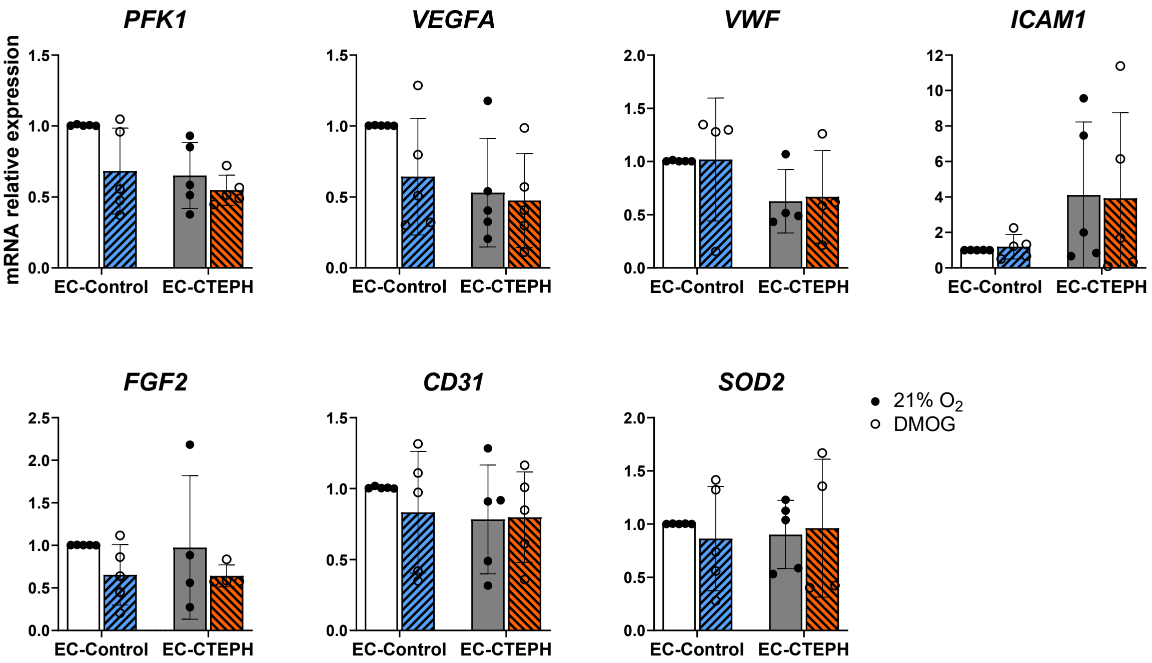

Supplementary Figure 3. Expression of different subsets of genes in EC-Control and EC-CTEPH after dimethyloxalylglycine (DMOG) treatment. (A) mRNA relative expression of metabolic gene *PFK1*, angiogenic genes *VEGFA, VWF, ICAM1, FGF2, CD31*; and oxidative stress gene *SOD2*, in five independent experiments with n=3 EC-Control at different passages and n=5 EC-CTEPH. Each data point represented the mean ± SD of the relative fold change with respect to EC-Control normoxia basal levels (21% O_2) normalized to reference gene ACTB (2-way ANOVA and Tukey's post-hoc test).

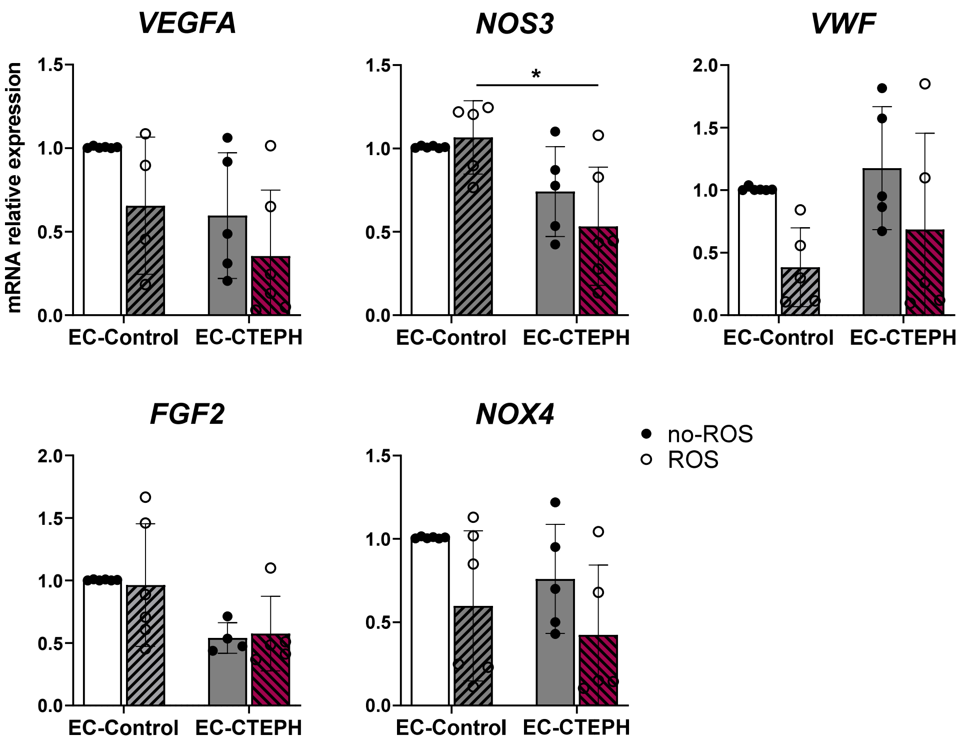

Supplementary Figure 4. Expression of different subsets of genes in EC-Control and EC-CTEPH after hydrogen peroxide (H_2O_2) treatment. mRNA relative expression of angiogenic genes *VEGFA, NOS3, VWF, FGF2*; and oxidative stress gene *NOX4* in 6 independent experiments with n=3 EC-Control at different passages and n=5 EC-CTEPH at different passages. Statistical significance was indicated as p<0.05*(2-way ANOVA and Tukey's post-hoc test). Each data point represented the mean ± SD of the relative fold change with respect to EC-Control normoxia basal levels (21% O_2) normalized to reference gene ACTB.

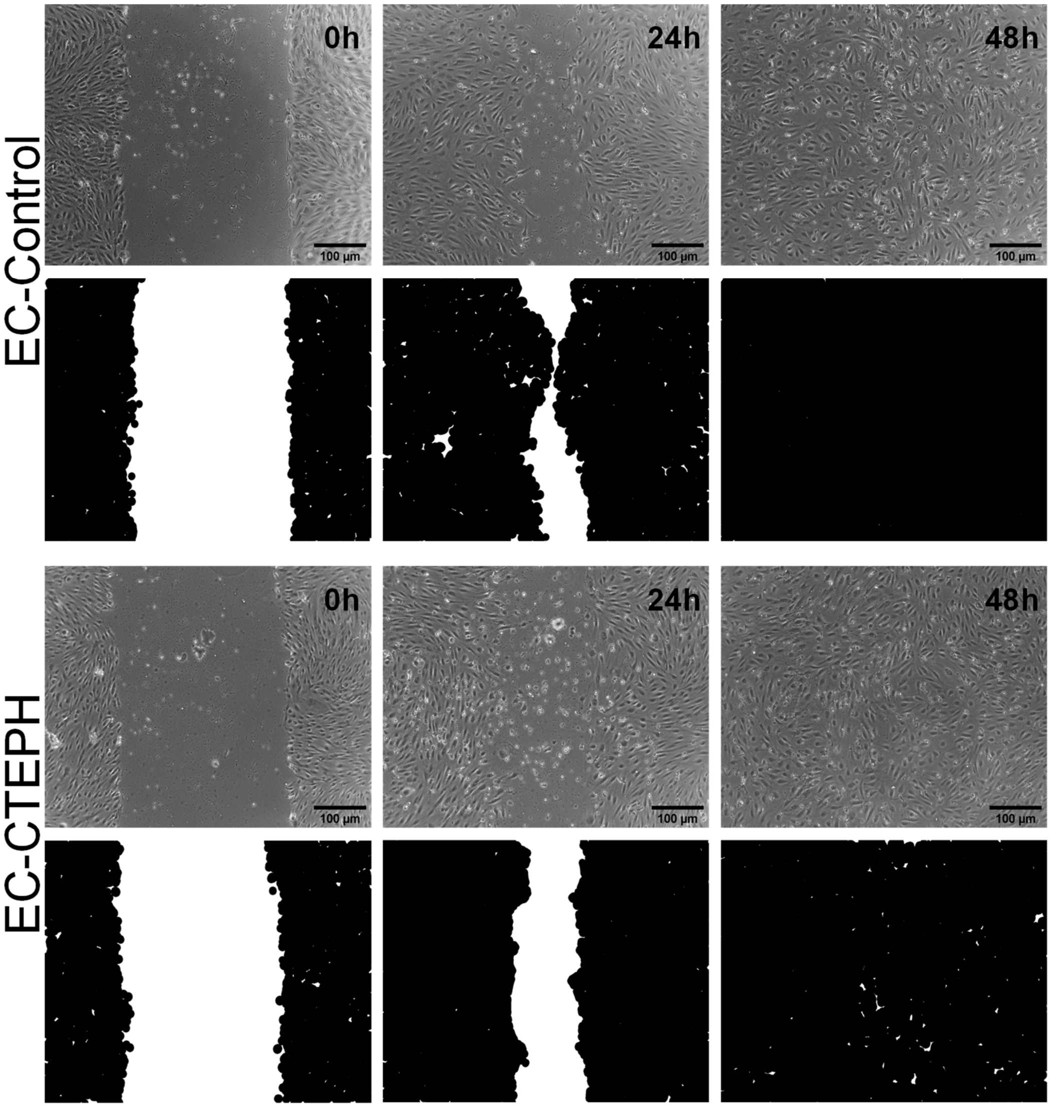

Supplementary Figure 5. Wound healing assay (WHA) images at 0h, 24h and 48h of both EC-Control and EC-CTEPH under hypoxia. Pictures were taken with a Zeiss Axiovert 200 at 10X magnification. Scale bar is 100µm.

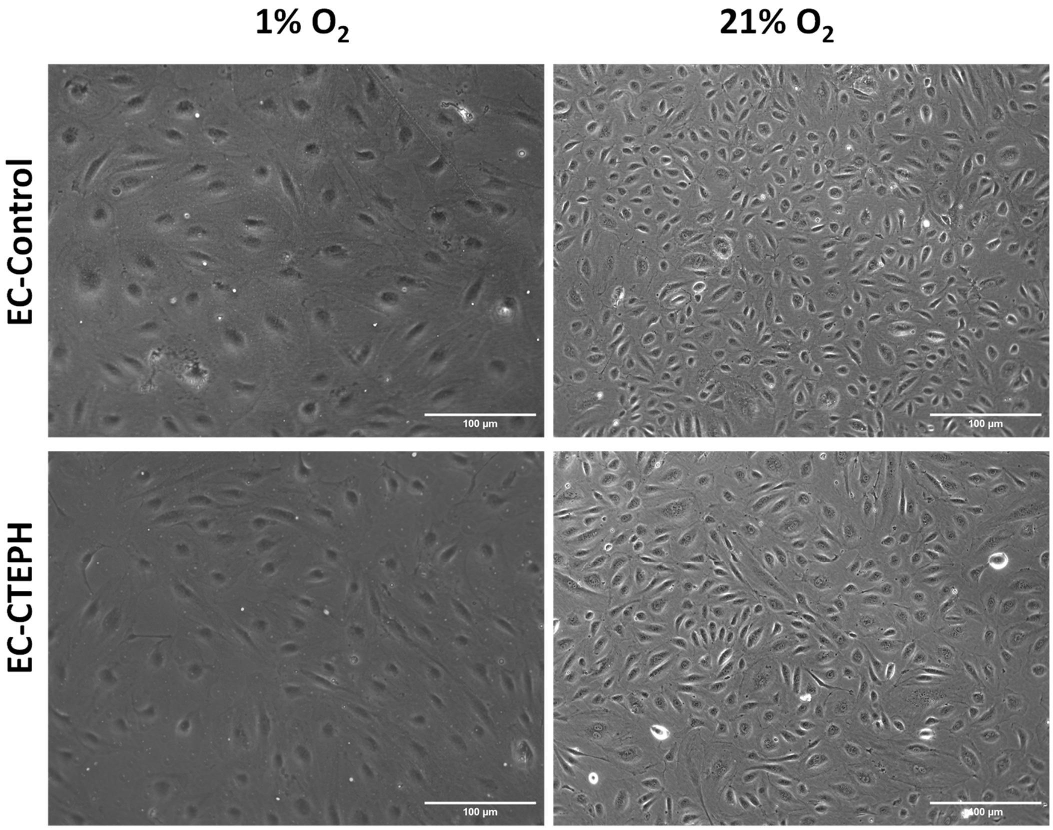

Supplementary Figure 6. Morphology of both EC-Control and EC-CTEPH at 1% O_2 and 21% O_2. Pictures were taken with a Zeiss Axiovert 200 at 20X magnification. Scale bar is 100µm.

	EC-CTEPH (n=5)	EC-Control (n=3)
Female/Male	5/0	1/2
Age years	70 ± 11	54 ± 18
BMI kg·m^{-2}	28.2 ± 4.2	
mPAP mmHg	36 ± 8.5	
PVR dyn·s·m^{-5}	563.8 ± 208.2	
Cardiac index L·min^{-1}·m^{-2}	2.4 ± 0.6	
Right atrial pressure mmHg	5.4 ± 6.5	
SvO2%	60.8 ± 7.0	
BNP pg·mL^{-1}	73.2 ± 88.2	
**WHO FC**		
I	0	
II	0	
III	5	
IV	0	

Supplementary Table S2. Hemodynamic parameters of samples used. n=5 EC-CTEPH and n=3 EC-Control samples were used. BMI stands for Body Mass Index, mPAP mean Pulmonary Artery Pressure, PVR Pulmonary Vascular Resistance, SvO_2 venous O_2 saturation, BNP Brain Natriuretic Peptide, WHO FC stands for functional class classification.

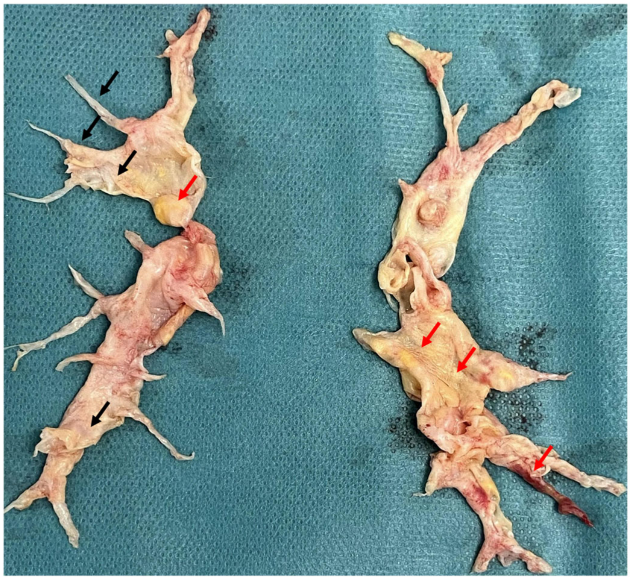

Supplementary Figure 7. Representative example of a pulmonary endarterectomy (PEA) specimen obtained from a patient with CTEPH. Black arrows indicate thrombus fragments

selected for endothelial cell isolation and culture. Red arrows indicate fibrotic areas that were discarded.

Primer	Forward sequence (5'-3')	Reverse sequence (5'-3')
ACTB	ACCCACACTGTGCCCATCTA	CGGAACCGCTCATTGCC
HK2	TCCCCTGCCACCAGACTA	TGGACTTGAATCCCTTGGTC
ENO1	CGTACCGCTTCCTTAGAA	GATGACACGAGGCTCACA
PDK1	GGTTACGGGACAGATGCAGT	CGTGGTTGGTGTTGTAATGC
LDHA	GCAGATTTGGCAGACAGTATAATG	GACATCATCCTTTATTCCGTAAAGA
PFK1	AGAGCGTTTCGATGATGCT	GTTGTAGGCAGCTCGGAGTC
VEGFA	CTACCTCCACCATGCCAAGT	GCAGTAGCTGCGCTGATAGA
NOS3	GGCCGGATCCAGTGGG	GTGGTTGCAGATGTAGGTGAACA
VWF	CCTTGAATCCCAGTGACCCTGA	GGTTCCGAGATGTCCTCCACAT
ICAM1	CAGAGGTTGAACCCCACAGT	CCTCTGGCTTCGTCAGAATC
FGF2	GAGCCCAGGAGTTCAAGACC	GAGACCACATGTACACGCCA
CD31	AAAGTCGGACAGTGGGACGT	GGCTGGGAGAGCATTTCACA
NOX4	CCGGCTGCATCAGTCTTAACC	TCGGCACAGTACAGGCACAA
SOD2	GCCCTGGAACCTCACATCAA	TCAGGTTGTTCACGTAGGCC
BNIP3	AGCGTCATGAAGAAAGGGG	ATCCGATGGCCAGCAAATGA
CASP8	GATTTGCTGATTACCTACCTAAACACT	TCTGAAATCTGATAGAGCATGACC
CASP9	ACACCCAGTGACATCTTTGTGT	GTCTCAACGTACCAGGAGCC
PAK1	CTGGAGACTCTCAGGGTCGAA	GGCGGATTAGGGCTTCCTC
TP53	GAGCTGAATGAGGCCTTGGA	CTGAGTCAGGCCCTTCTGTCTT
EGLN1	CCC TCA TGA AGT ACA ACC AGC AT	CAT CTG CAT CAA AAT ACC AAA CAG T
EGLN3	GCC GGC TGG GCA AAT ACT A	CCG GAT AGC AAG CCA CCA T
HIF1AN	CCTCTAACCTGCTGCTCATTGG	GTAGAGGCACTCGAACTGATCC

Supplementary Table S3. Primers used for this study. Forward and Reverse sequences of the different primers used in this study. *ACTB* was used as a housekeeping gene.

Name	Company	Code	Characteristics	Host	Reactivity	kDa
LDHA	Santa Cruz	sc-137243	monoclonal	mouse	human	35
eNOS	Abcam	ab76198	monoclonal	mouse	human	133
PDK1	Enzo Life Sciences, Inc	ADI-KAP-PK112	polyclonal	rabbit	human	48
HIF-2α	Abcam	ab207607	monoclonal	rabbit	human	118
HIF-1α	BD Transduction Laboratories	610959	monoclonal	mouse	human	120
VINCULIN	Abcam	ab129002	monoclonal	rabbit	human	124
β-ACTIN	Sigma-Aldrich	A3854	monoclonal	mouse	human	42

Supplementary Table S4. Antibodies used for this study.

a) Figure 5D. Ponceau Red Staining

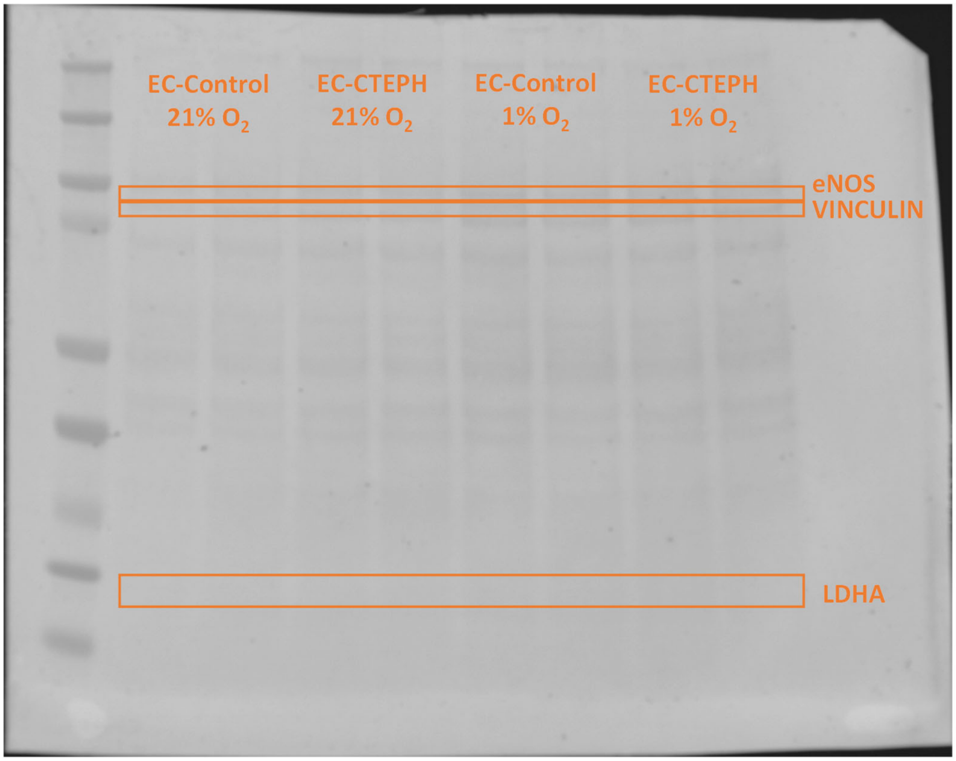

b) Figure 5D. LDHA

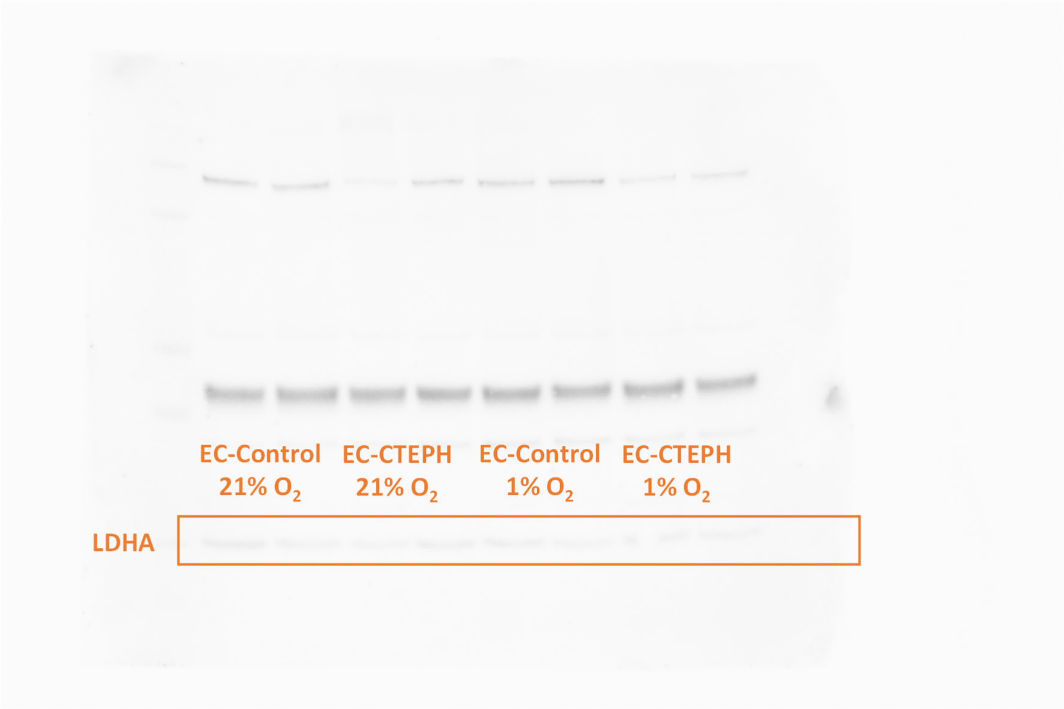

c) Figure 5D. eNOS

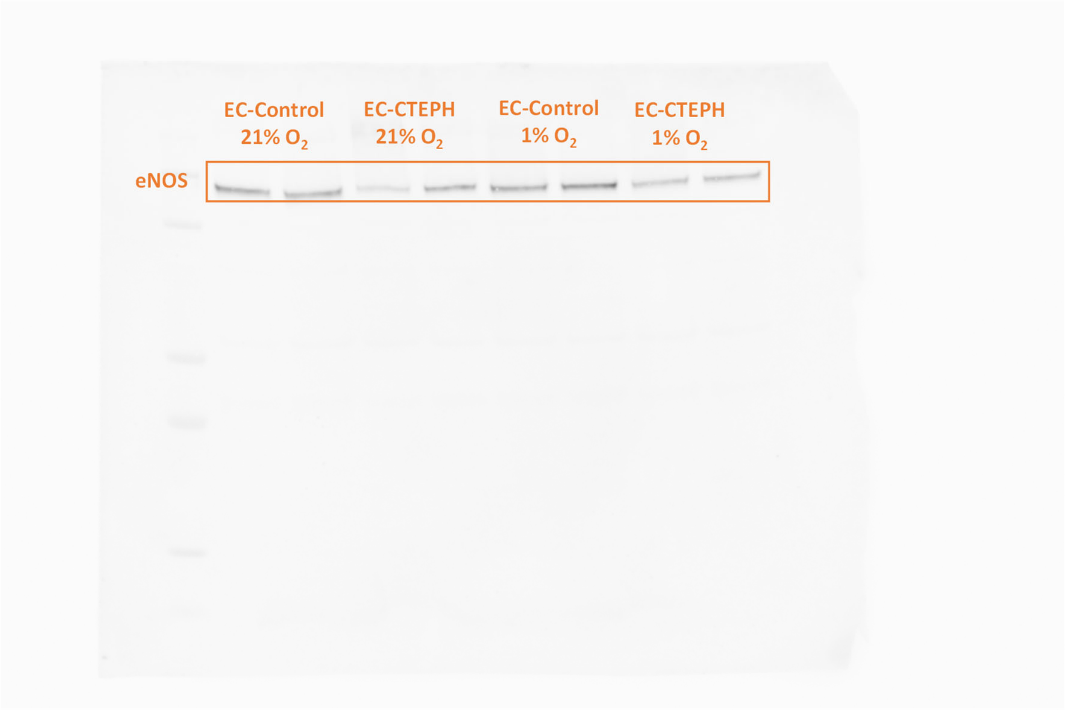

d) Figure 5D. Vinculin

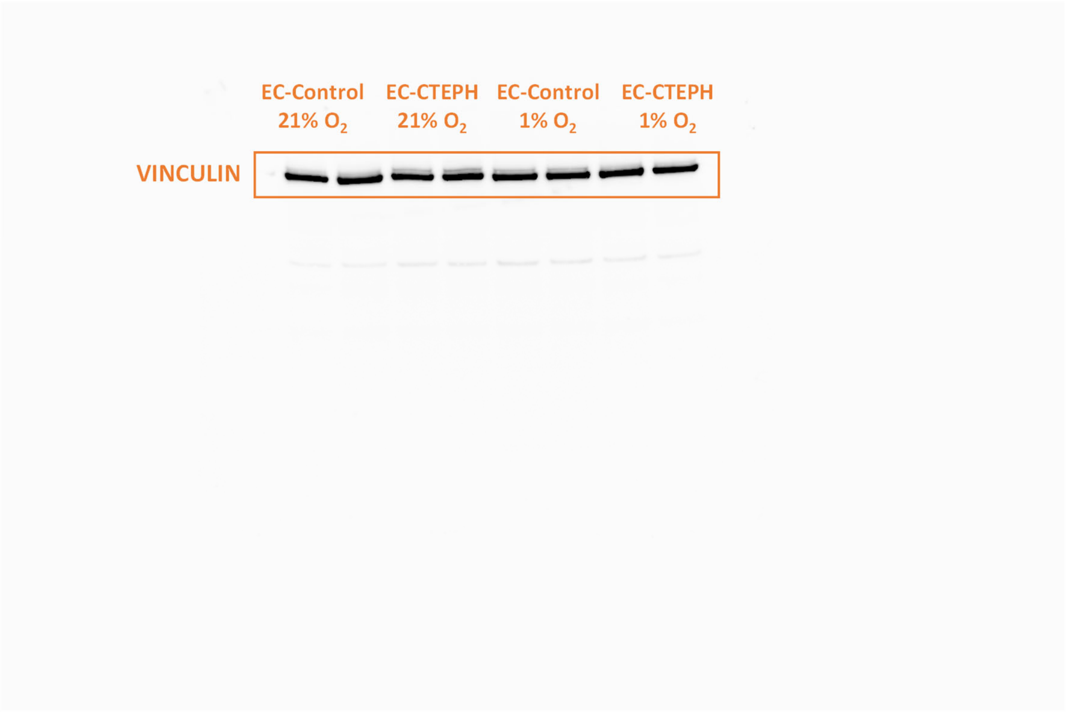

e) Figure 5D. Ponceau Red staining membrane PDK1 and β-ACTIN

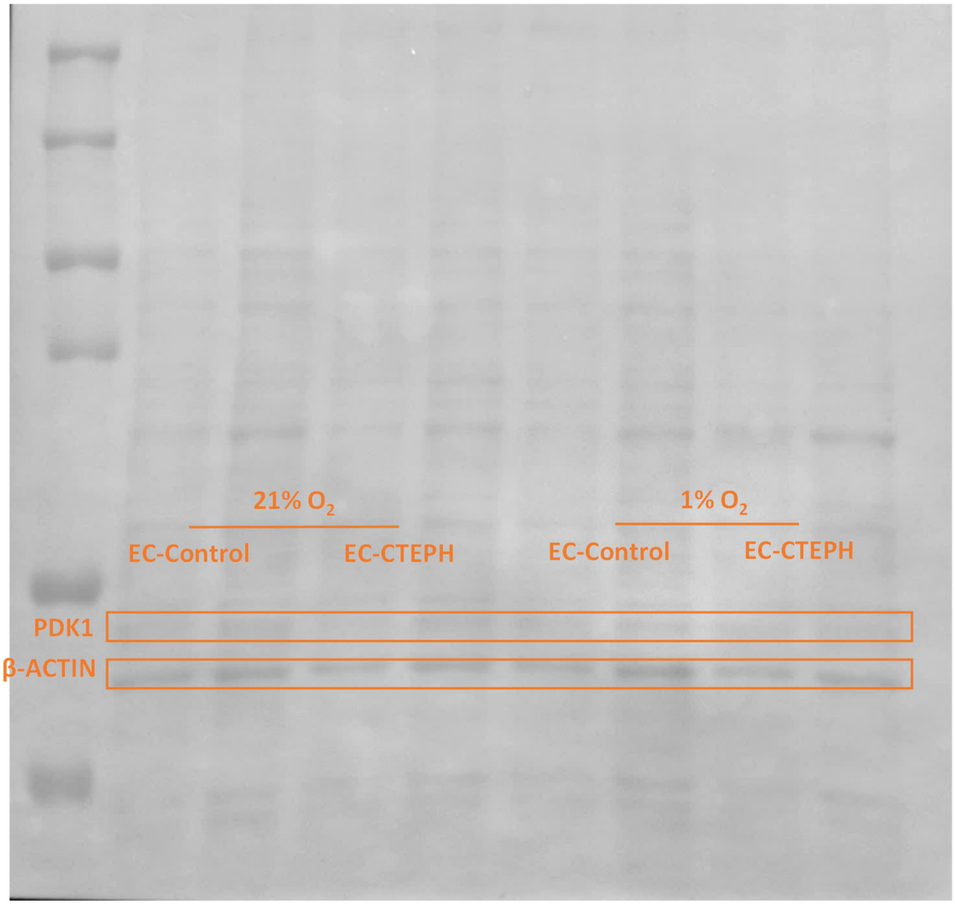

f) Figure 5D. Cropped blot PDK1

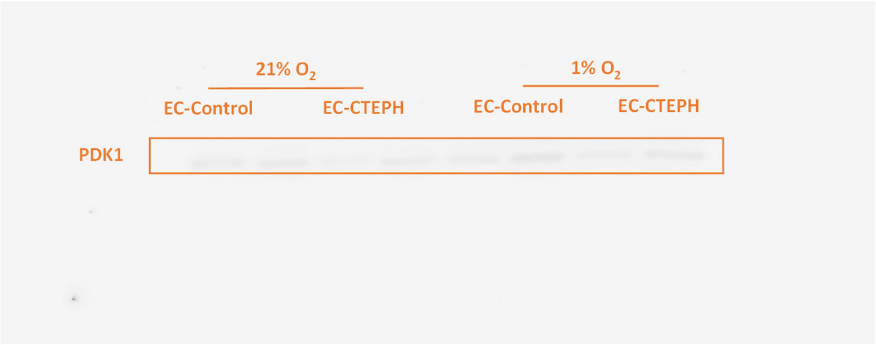

g) Figure 5D. Cropped blot β-ACTIN

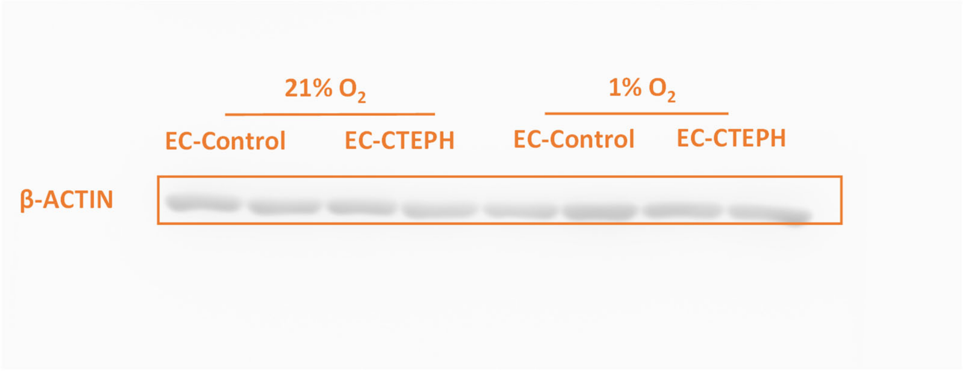

Supplementary Figure 8. Full-length membrane and blots represented in Figure 5 in order of appearance.

a) Figure 7B. Ponceau Red staining HIF-1α, HIF-2α and β-ACTIN

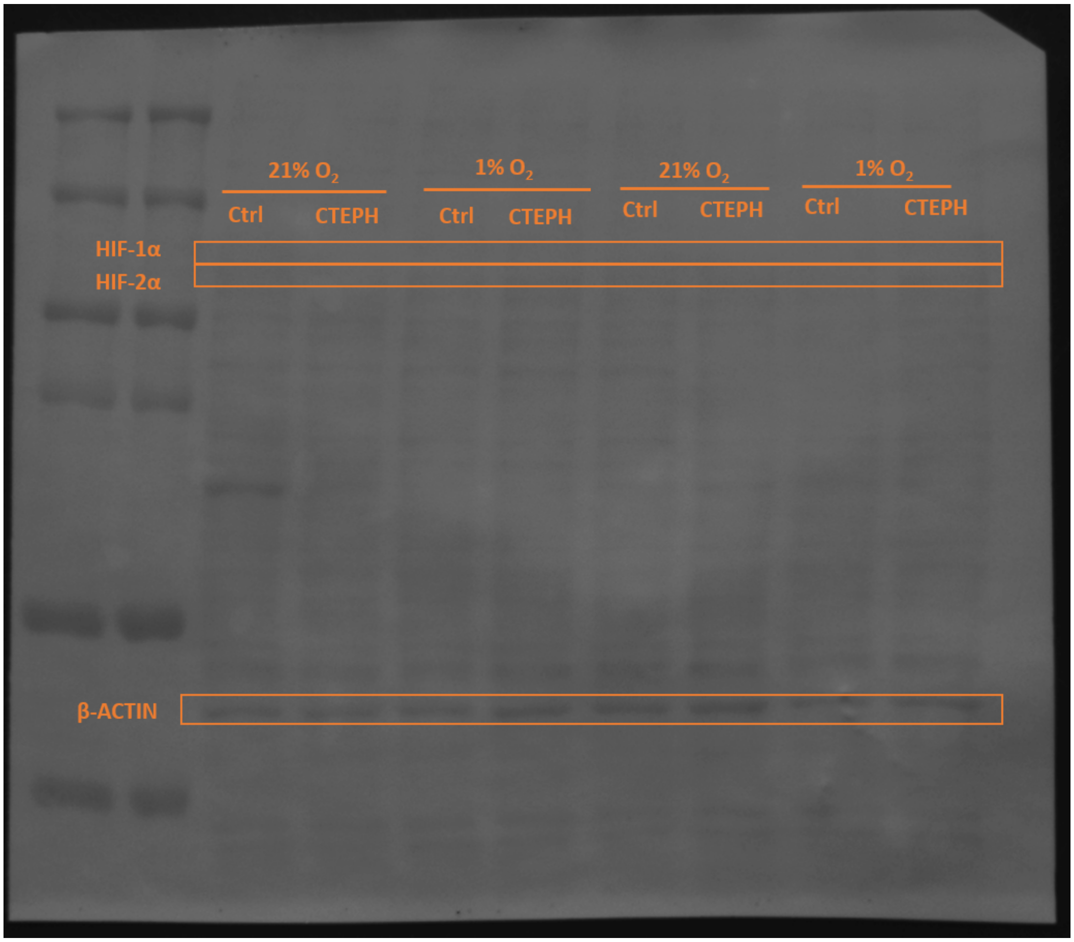

b) Figure 7B. HIF-2α

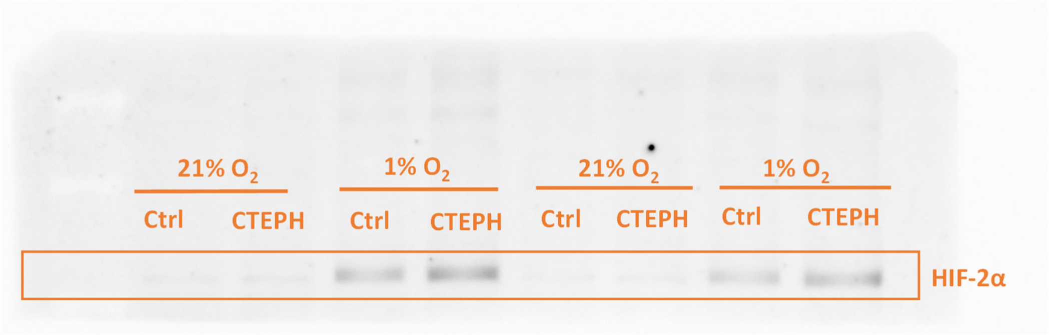

c) Figure 7B. HIF-1α

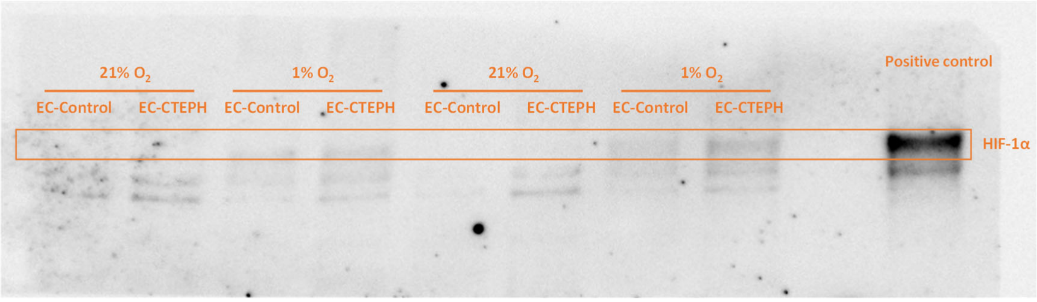

d) Figure 7B. β-ACTIN

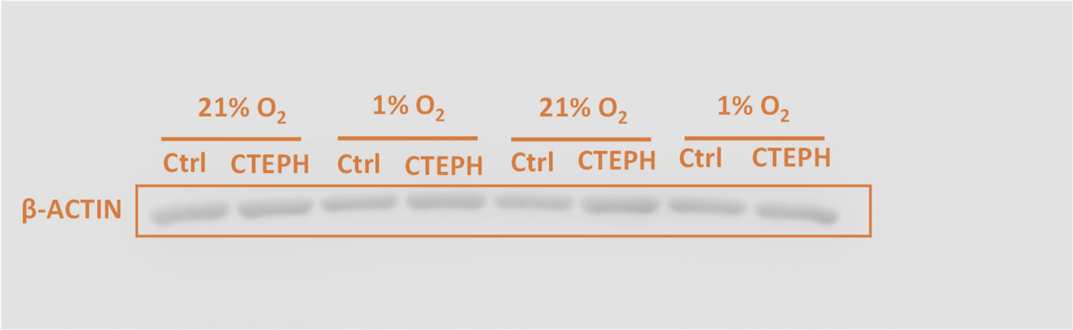

Supplementary Figure 9. Full-length membranes and blots represented in Figure 7 in order of appearance.

	Group comparison	Mean difference	P-value	Significance
**0h**	EC-Control 21% O_2 vs 1% O_2	0,000	>0,999	ns
	EC-CTEPH 21% O_2 vs 1% O_2	0,000	>0,999	ns
	EC-Control 21% O_2 vs EC-CTEPH 21% O_2	0,000	>0,999	ns
	EC-Control 1% O_2 vs EC-CTEPH 1% O_2	0,000	>0,999	ns
**6h**	EC-Control 21% O_2 vs 1% O_2	-8,190	0,478	ns
	EC-CTEPH 21% O_2 vs 1% O_2	-4,770	0,882	ns
	EC-Control 21% O_2 vs EC-CTEPH 21% O_2	-4,152	0,885	ns
	EC-Control 1% O_2 vs EC-CTEPH 1% O_2	-0,732	1,000	ns
**8h**	EC-Control 21% O_2 vs 1% O_2	-4,340	0,887	ns
	EC-CTEPH 21% O_2 vs 1% O_2	-8,837	0,580	ns
	EC-Control 21% O_2 vs EC-CTEPH 21% O_2	2,787	0,973	ns
	EC-Control 1% O_2 vs EC-CTEPH 1% O_2	-1,711	0,994	ns
**10h**	EC-Control 21% O_2 vs 1% O_2	-9,040	0,390	ns
	EC-CTEPH 21% O_2 vs 1% O_2	-8,483	0,612	ns
	EC-Control 21% O_2 vs EC-CTEPH 21% O_2	-0,194	>0,999	ns
	EC-Control 1% O_2 vs EC-CTEPH 1% O_2	0,363	>0,999	ns
**24h**	EC-Control 21% O_2 vs 1% O_2	11,640	0,219	ns
	EC-CTEPH 21% O_2 vs 1% O_2	24,660	0,0002	***
	EC-Control 21% O_2 vs EC-CTEPH 21% O_2	8,923	0,402	ns
	EC-Control 1% O_2 vs EC-CTEPH 1% O_2	21,940	0,002	**
**30h**	EC-Control 21% O_2 vs 1% O_2	12,45	0,281	ns
	EC-CTEPH 21% O_2 vs 1% O_2	8,74	0,589	ns
	EC-Control 21% O_2 vs EC-CTEPH 21% O_2	-3,837	0,945	ns
	EC-Control 1% O_2 vs EC-CTEPH 1% O_2	-7,548	0,696	ns
**48h**	EC-Control 21% O_2 vs 1% O_2	0,000	>0,999	ns
	EC-CTEPH 21% O_2 vs 1% O_2	0,000	>0,999	ns
	EC-Control 21% O_2 vs EC-CTEPH 21% O_2	0,000	>0,999	ns
	EC-Control 1% O_2 vs EC-CTEPH 1% O_2	0,000	>0,999	ns

Supplementary Table S5. Statistical analysis details of wound healing assay experiments. This table shows multiple comparisons performed using 2-way ANOVA and Tukey’s post-hoc test. Statistical significance was indicated as $p<0.01$**, $p<0.001$***. Data were obtained from six independent experiments (n=3 EC-Control, n=5 EC-CTEPH, passages 4-6).
